# Identification of the Potential Molecular Mechanism of *TGFBI* Gene in Persistent Atrial Fibrillation

**DOI:** 10.1155/2022/1643674

**Published:** 2022-11-08

**Authors:** Yao-Zong Guan, Hao Liu, Huan-Jie Huang, Dong-Yan Liang, Si-Ying Wu, Tang Zhang

**Affiliations:** ^1^Department of Cardiology, Second Affiliated Hospital of Guangxi Medical University, Nanning, 530007 Guangxi, China; ^2^Cardiac Rehabilitation Center, Second Affiliated Hospital of Guangxi Medical University, Nanning, 530007 Guangxi, China

## Abstract

**Background:**

Transforming growth factor beta-induced protein (TGFBI, encoded by *TGFBI* gene), is an extracellular matrix protein, widely expressed in variety of tissues. It binds to collagens type I, II, and IV and plays important roles in the interactions of cell with cell, collagen, and matrix. It has been reported to be associated with myocardial fibrosis, and the latter is an important pathophysiologyical basis of atrial fibrillation (AF). However, the mechanism of TGFBI in AF remains unclear. We aimed to detect the potential mechanism of *TGFBI* in AF via bioinformatics analysis.

**Methods:**

The microarray dataset of GSE115574 was examined to detect the genes coexpressed with *TGFBI* from 14 left atrial tissue samples of AF patients. *TGFBI* coexpression genes were then screened using the R package. Using online analytical tools, we determined the Kyoto Encyclopedia of Genes and Genomes (KEGG) pathway enrichment analysis, Gene Ontology (GO) annotation, and protein-protein interaction (PPI) network of *TGFBI* and its coexpression genes. The modules and hub genes of the PPI-network were then identified. Another dataset, GSE79768 was examined to verify the hub genes. DrugBank was used to detect the potential target drugs.

**Results:**

In GSE115574 dataset, a total of 1818 coexpression genes (769 positive and 1049 negative) were identified, enriched in 120 biological processes (BP), 38 cellular components (CC), 36 molecular functions (MF), and 39 KEGG pathways. A PPI-network with average 12.2-degree nodes was constructed. The genes clustered in the top module constructed from this network mainly play a role in PI3K-Akt signaling pathway, viral myocarditis, inflammatory bowel disease, and platelet activation. *CXCL12*, *C3*, *FN1*, *COL1A2*, *ACTB*, *VCAM1*, and *MMP2* were identified and finally verified as the hub genes, mainly enriched in pathways like leukocyte transendothelial migration, PI3K-Akt signaling pathway, viral myocarditis, rheumatoid arthritis, and platelet activation. Pegcetacoplan, ocriplasmin, and carvedilol were the potential target drugs.

**Conclusions:**

We used microdataset to identify the potential functions and mechanisms of the *TGFBI* and its coexpression genes in AF patients. Our findings suggest that *CXCL12*, *C3*, *FN1*, *COL1A2*, *ACTB*, *VCAM1*, and *MMP2* may be the hub genes.

## 1. Introduction

Atrial fibrillation (AF) is a complex arrhythmia, which makes about 5.2 million people suffering palpitations in the US until 2010 and would increase to 12.1 million in 2030 [1], owning to 25-30% of ischemic stroke [2]. In addition, persistent atrial fibrillation leads to mitral regurgitation and decreases up to 25% of left heart ejection function, aggravating heart failure even results in death [3]. It is estimated that around 6-12 million people will suffer this condition in the US by 2050 and 17.9 million people in Europe by 2060 [4, 5]. In China, it is estimated that the lifetime risk of AF is approximately 1 to 5 [6]. Populations of AF bring great economic burden to patients and countries worldwide. Data from previous researches shows that, in 18-45 years old AF patients, the mean cost of AF management in hospitalization was $7924 in 2015 [7]. To make matters worse, the prevalence of AF and the economic burden is still at a high level. To date, AF is considered as a disorder of electrical activity in the atrium, and the drive center probably locates in the left atrium-pulmonary vein junction. Although catheter ablation and balloon cryoablation based on circumpulmonary vein electrical dissection isolation show an exciting effect, but the mechanism of AF remains unclear.

Transforming growth factor beta-induced protein (*TGFBI*, HGNC: 11771, Ensembl: ENSG00000120708, MIM: 601692), also known as CSD, CDB1, EBMD, and CDGG1, encodes an RGD-containing protein that binds to type I, II, and IV collagens. In an early study, Li et al. [8] reported that the expression of *TGFBI* can be upregulated by stimulated by TGF-beta, activating the TGF-beta BMP signaling pathway and induced the differentiation of bone marrow stem cells into immature cardiomyocytes. A recent research confirmed that *TGFBI* is a candidate marker for human cardiac fibroblasts in vivo and in vitro [9]. In addition, Chen et al. [10] reported that *TGFBI* is a target of microRNA-21, which plays a role in the regulation of fibrosis. In miR-21 knockdown cells, *TGFBI* was significantly upregulated, which promoted the formation of fibrosis. It is well known that AF is an age-related disease, and atrial fibrosis has emerged as an important pathophysiological contributor in aging, and has been linked to recurrences and complications of AF [11]. Thus, *TGFBI* is likely to play a role in the process of AF. However, little is known of the clear mechanism of *TGFBI* in AF.

In the near decades, microarray-sequencing technology has rapidly developed and has significantly promoted the improvement of basic and clinical medicine. The Gene Expression Omnibus (GEO) database is a large repository, integrated with a series of high-throughput microarray and next-generation sequence functional genomic datasets, and is free for global researchers [12]. Up to now, GEO database have helped numerous researchers to identify key mechanisms and hub targets of cardiovascular diseases, tumors, and other diseases [13–15]. The regulation of a pathway often involves several genes, and there are coexpression relationships among these genes. In the current study, we aimed to further understand the mechanism of *TGFBI* in AF patients by detecting the *TGFBI* and its coexpression genes and their pathways enriched in AF patients.

## 2. Materials and Methods

### 2.1. Study Design and Dataset Selection

Genes with similar functions or involved in the same pathways often have coexpression relationships. In order to detect the mechanism of *TGFBI* in AF, we aimed to screen out the genes with coexpression relationship to *TGFBI* for analysis. Indeed, it was similar to the analysis of the subanalysis of the coexpression of blocks in weighted correlation network analysis (WGCNA) [16]. The difference was that WGCNA screens coexpression from differentially expressed genes or RNAs, but the central idea of our study was to take *TGFBI* as the central gene and combine its coexpressed genes to detect their biological functions, cellular localization, and enrichment pathways in AF. The cor function in R package can be used to detect the correlation coefficient between two variables (https://stat.ethz.ch/R-manual/R-devel/library/stats/html/cor.html). The code for the cor function is as follows: cor(*x*, *y* = NULL, use = “everything,” method = c (“pearson,” “kendall,” “spearman”)). The annotation information of the code was as follows: *x*: a numeric vector, matrix, or data frame; *y*: NULL (default) or a vector, matrix, or data frame with compatible dimensions to *x*. The default is equivalent to *y* = *x* (but more efficient); use an optional character string giving a method for computing covariances in the presence of missing values. This must be (an abbreviation of) one of the strings “everything,” “all.obs,” “complete.obs,” “na.or.complete,” or “pairwise.complete.obs”; method: a character string indicating which correlation coefficient (or covariance) is to be computed. One of “pearson” (default), “kendall,” or “spearman” can be abbreviated. In prior study, we used this method to obtain the mechanism of *NPPB* and its coexpressed genes in different patients with heart failure [17]. Zhang et al. [18] also used cor function to analysed the correlation ship between the methylation levels and expression levels of the differentially methylated protein-coding genes for construction a nomogram survival model of the lung squamous cell carcinoma.

**Figure 1:**
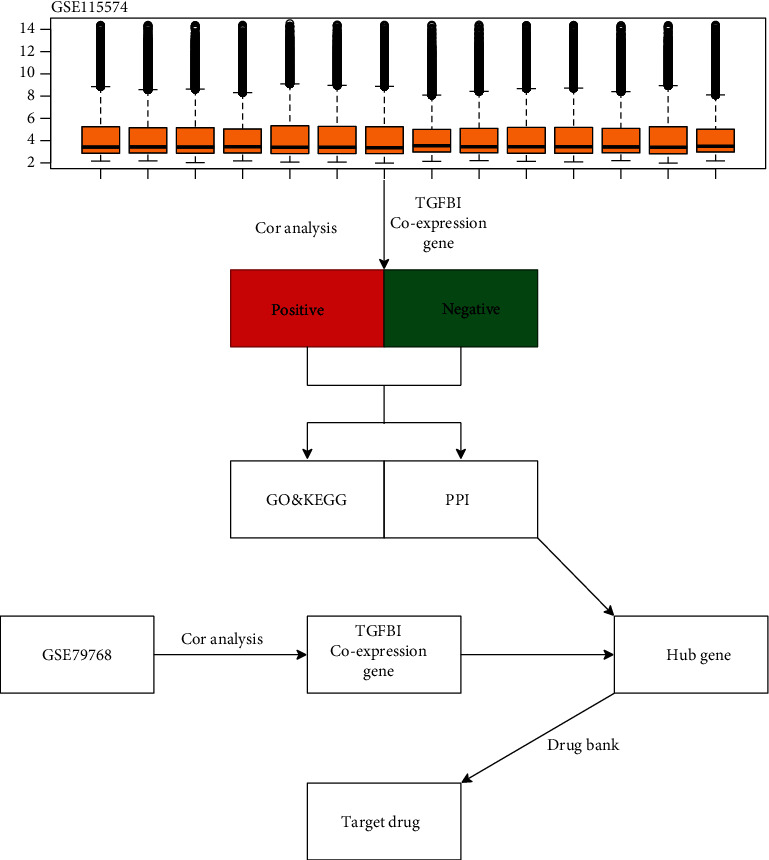
Flowchart of the current study. Orange box presents the expression of genes in every sample of GSE114457. DAVID, the Database for Annotation, Visualization, and Integrated Discovery.

We selected one microarray dataset for analysis, and another for validation. The microarray dataset GSE115574 was retrieved from the GEO database. This dataset contained 14 left atrial tissue samples from persistent AF patients [19]. The expression of all genes in every sample is shown in Figure 1, and no significant outlier samples were found. Therefore, the included samples could be used for further analysis. Another dataset GSE79768, containing 7 left atrial specimens from persistent AF patients, was retrieved from the GEO database for validation. The flowchart is shown in Figure 1. The further analysis were performed via the software RStudio (based on R package, version 3.6.4), on the platform of Windows 10 system (64-bit).

### 2.2. Identification of TGFBI Coexpression Genes and Pathway Enrichment Analyses

A screening of coexpression genes for *TGFBI* from the samples was performed by the cor function in R (version 3.6.4). In our study, the code was set as: cor(*x*, *y*, method = pearson), *x* and *y* represent the expression levels of *TGFBI* and other genes, respectively. The strength of the correlation was represented by the calculated correlation coefficient. Screening criteria were as follows: *P* < 0.05 and |Pearson correlation coefficient| ≥ 0.6. The online database, the Database for Annotation, Visualization, and Integrated Discovery (DAVID, version 6.8), was used for GO and KEGG enrichment analyses [20–22]. *P* value of <0.05 was set as significance. The ggplot2 package was used for visualization of the results in R (version 3.6.4).

### 2.3. Integration of the PPI-Network

The STRING (version 10.5) database was used for evaluating the interactions among the coexpression genes, and a combined interaction score of > 0.4 was set as significant [23]. In addition, the top 10 hub genes were identified used Cytoscape plugin cytoHubba (version 0.1) with the degree ratio ranking method. Furthermore, the MCODE and ClueGO apps in Cytoscape were used to identify the modules, namely the GO annotation and KEGG pathway enrichment analyses, respectively, of the PPI-network [24].

## 3. Results

### 3.1. Identification of TGFBI Coexpression Genes and Pathway Enrichment Analyses

**Figure 2:**
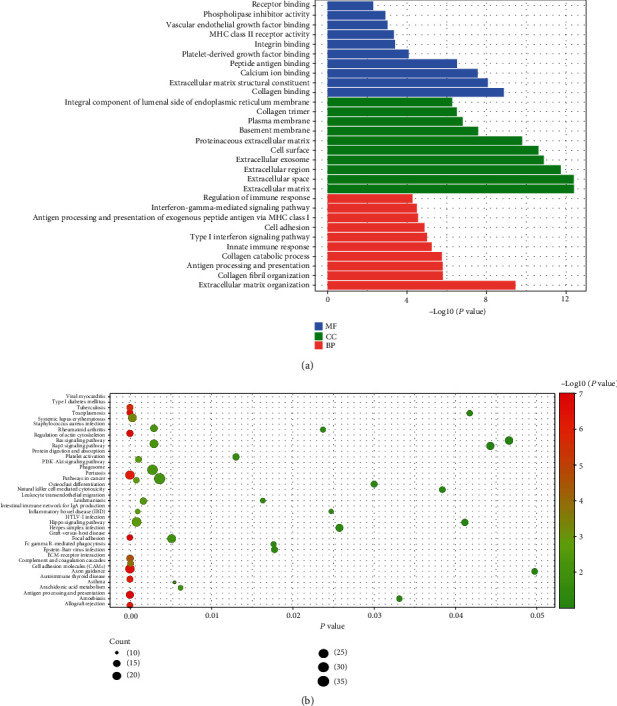
Go annotation and KEGG pathway of *TGFBI* and its coexpression genes in atrial fibrillation. (a) Go annotation: the strip represents the value of −log10(*P*) of GO term. (b) KEGG pathway: the size of ball represents the count of enriched genes, and the color of ball represents the value of −log10(*P*) of pathway.

A total of 1049 negatively coexpressed genes and 769 positively coexpressed genes were identified after cor analysis. These genes were enriched in a series of BPs, such as collagen fibril organization (GO:0030199), collagen catabolic process (GO:0030574), cell adhesion (GO:0007155), and angiogenesis (GO:0001525); 38 CCs, such as collagen trimer (GO:0005581), proteinaceous extracellular matrix (GO:0005578), and basement membrane (GO:0005604); 36 MFs, such as collagen binding (GO:0005518), receptor binding (GO:0005102), and peptide antigen binding (GO:0042605). As shown in Figure 2(a), the top 30 GOs were selected for visualization, and the whole information of the GO results were shown in Table S1.

In addition, a total of 39 KEGG pathways were identified, such as antigen processing and presentation (hsa04612), cell adhesion molecules (CAMs, hsa04514), viral myocarditis (hsa05416), and PI3K-Akt signaling pathway (hsa04151). The visualization of these KEGG pathways is shown in Figure 2(b), and the whole information of these pathways is shown in Table 1.

### 3.2. PPI-Network Construction and Hub Gene Identification

**Figure 3:**
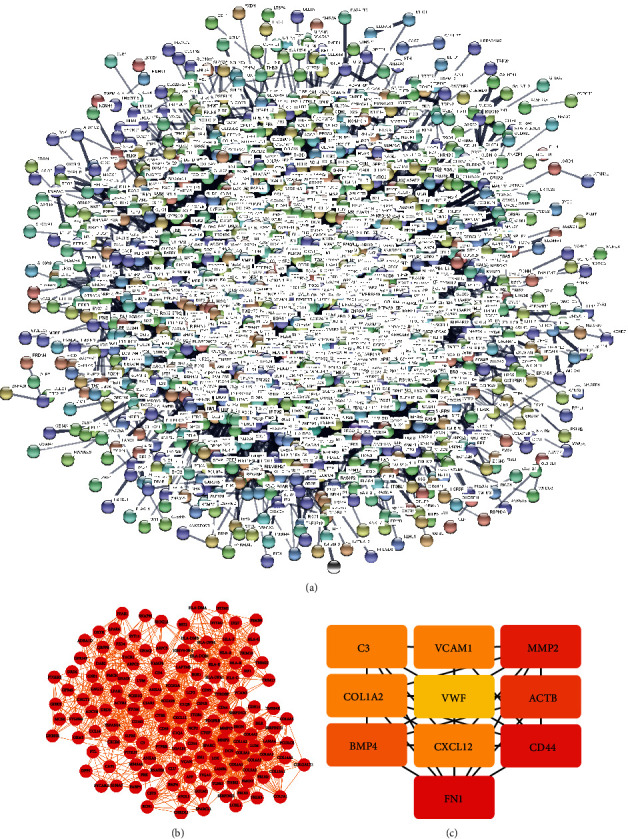
PPI-network of *TGFBI* and its coexpression genes in atrial fibrillation and hub genes. (a) PPI-network constructed based on *TGFBI* and its coexpression genes. (b) Cluster 1 of the PPI-network: the red ball represents the coexpression, and the orange line represents the correlation. (c) Hub genes: the color depth represents the ranking of hub genes. The sequence of colors is red-orange-yellow from high to low ranking.

As shown in Figure 3(a), the interactions between *TGFBI* and its coexpression genes were presented by a PPI-network with 1465 nodes. The average node degree was 12.2, and the PPI enrichment *P* value was <1.0E-16. This finding was saved in *TSV* format and then imported into Cytoscape for visualization. With a cutoff criterion of a degree that is >5 and a K-core >5, three clusters were constructed as shown in Figure S1; and it is shown in Figure 3(c) that the top 10 hub genes of this PPI-network were also identified (*CXCL12*, *C3*, *FN1*, *COL1A2*, *ACTB*, *VCAM1*, *MMP2*, *VWF*, *BMP4*, and *CD44*), with the degree ratio ranking method.

**Figure 4:**
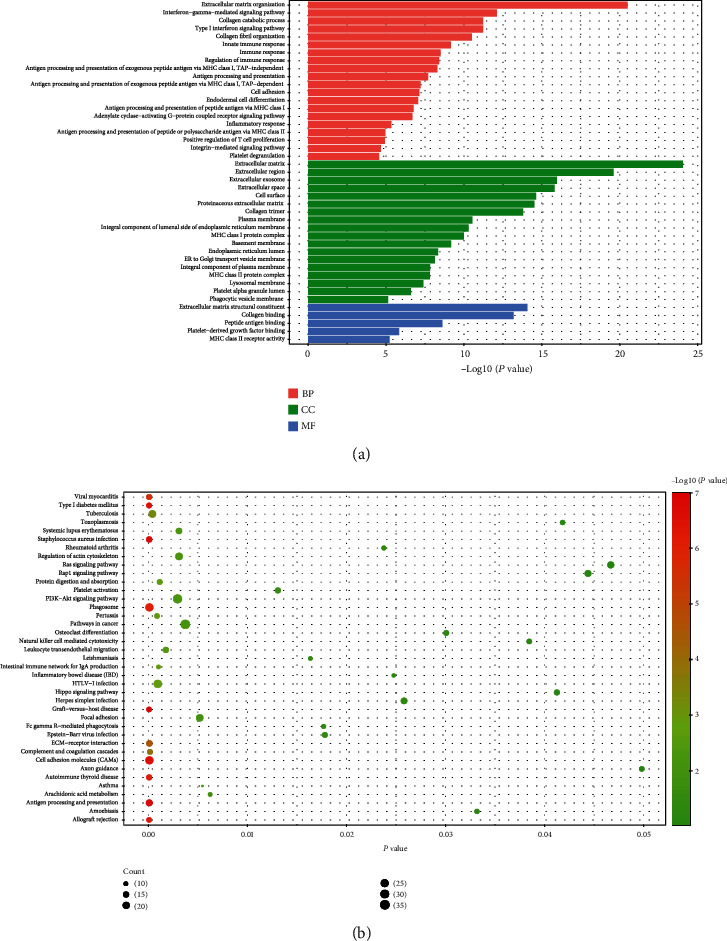
Go annotation and KEGG pathway of TGFBI and its coexpression genes in cluster 1. (a) Go annotation: the strip represents the value of −log10(*P*) of GO term. (b) KEGG pathway: the size of ball represents the count of enriched genes, and the color of ball represents the value of −log10(*P*) of pathway.

We selected the first cluster, which is descripted in Figure 3(b) for GO and KEGG pathway analyses and found that the coexpression genes in this cluster enriched in 20 BPs, such as collagen catabolic process (GO:0030574), collagen fibril organization (GO:0030199), and endodermal cell differentiation (GO:0035987); 18CCs, such as collagen trimer (GO:0005581), plasma membrane (GO:0005886), and platelet alpha granule lumen (GO:0031093); 5MFs, such as collagen binding (GO:0005518), and platelet-derived growth factor binding (GO:0048407), and 47 KEGG pathways like viral myocarditis (hsa05416), PI3K-Akt signaling pathway (hsa04151), rheumatoid arthritis (hsa05323), and Inflammatory bowel disease (IBD, hsa05321). The visualization of the mentioned GOs and KEGG pathways is shown in Figure 4, and the whole information of them is shown in Table S2.

### 3.3. Verification of the Hub Gene

The correlations between the hub genes and *TGFBI* were verified in GSE79768 dataset. We used cor analysis to detect the correlation value between the coexpression gene and *TGFBI* in GSE79768. As shown in Table 2, except for *VWF*, *BMP4*, and *CD44*, the correlations between *TGFBI* with *CXCL12*, *C3*, *FN1*, *COL1A2*, *ACTB*, *VCAM1*, and *MMP2* were consistent with the results in GSE115574 dataset as they were all positively correlative and the *P* value <0.05.

### 3.4. Pathways and the Verified Hub Genes

**Figure 5:**
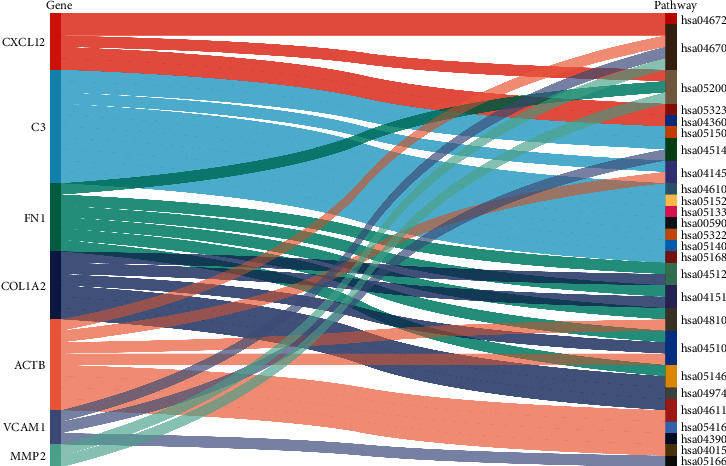
Sankey diagram of the hub genes and the KEGG pathways they enriched in. The band on the left represents the core gene; and the band on the right represents the pathway the hub gene enriched in. The width of the cloth connecting the two strips represents the *P* value.

It is descripted in Figure 5, the verified hub genes were enriched in different and/or same KEGG pathways. *CXCL12*, *ACTB*, and *MMP2* were enriched in leukocyte transendothelial migration (hsa04670). *FN1* and *COL1A2* were both enriched in PI3K-Akt signaling pathway (hsa04151), amoebiasis (hsa05146), and focal adhesion (hsa04510). In addition, *CXCL12* was enriched in rheumatoid arthritis (hsa05323), and *C3* was enriched in leishmaniasis (hsa05140).

### 3.5. Potential Drugs Targeted by the Verified Hub Genes

To detect the potential drugs of the hub genes, we used the online database DrugBank (http://www.drugbank.ca) to identify the drug that targeted by the verified hub gene. DrugBank is an online and free-access database, integrating the mechanisms, targets, and interactions of the drugs [25]. We selected the genes, which have a complete record of actions and have gotten the approval for presentation in Table 3.

## 4. Discussion

The rapid development of sequencing technology has helped researchers to gain a deeper understanding of several comprehensive diseases, such as cardiovascular diseases, tumors, and autoimmune diseases. The mechanism of AF remains not well clear. In the current study, we identified the potential mechanism of *TGFBI* and its coexpression genes in AF patients, and verified the hub genes, hoping to provide reference for the further study of AF.

**Figure 6:**
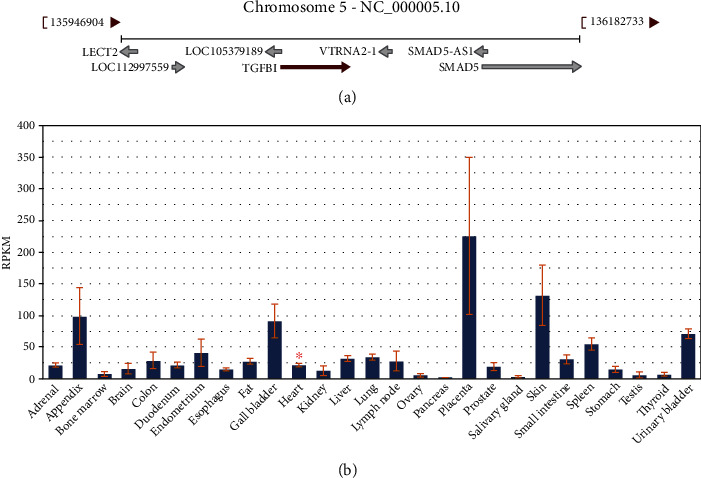
Location and expression about *TGFBI*. (a) Location of *TGFBI* in chromosome. (b) Expression of *TGFBI* in different samples.

It is descripted in Figure 6 that *TGFBI* is located in 5q31.1, and it expresses in several organs like heart, liver, colon, urinary bladder, and so on (https://www.ncbi.nlm.nih.gov/gene/7045). In early studies, the research of TGFBI mainly focused on corneal dystrophy as it is a primary disease-causing gene of corneal dystrophy, leading to protein deposits on the cornea then cause blindness [26, 27]. In addition, it is also a star gene owing to its important effect on the outcome of cancer and the sensitivity to chemotherapeutic drugs. It affects the progress of tumor main because it would promote cell proliferation, migration, and change the microenvironment [28, 29]. However, the function of TGFBI in heart remains unclear. An early study suggested that in diseased heart, the expression of *TGFBI* mRNA induced [30]. Similarly, Schwanekamp et al. [31] reported that the expression of TGFBI in the heart increased after injury. What is more, in a plasma proteome profiling, TGFBI has a potential role in extracellular matrix remodeling in fibrosis [32]. A recent study further confirmed that TGFBI and ADAM19 were associated with the TGF-*β*1 pathway and cardiac fibrosis [33]. Thus, *TGFBI* probably plays a role in myocardial fibrosis.

Myocardial fibrosis is a key pathophysiological mechanism of heart failure and arrhythmia. Cardiac fibrosis gets the pathological feature with disorder of cardiac muscle cells and notable increasing of collagen fibers. Disordered myocardium and interwoven collagen fibers lead to the uncoordinated conduction of power induced by systole and diastole, and anisotropy of action potentials, which lead to the decrease of heart function and the risk of arrhythmias [34, 35]. As is known, genes with similar functions often show coexpression relationships and then coregulate biological functions. We found that *TGFBI* and its coexpression genes enriched in several GOs and pathways like collagen fibril organization, PI3K-Akt pathway, and viral myocarditis. PI3K-Akt pathway has been reported regulating myocardial fibrosis. *FN1* and *COL1A2* genes are both enriched in this pathway. Huang et al. [36] reported that miR-144-3p/FN1 and miR-9-3p/FN1 pathways may play an important role in myocardial fibrosis. In addition, *FN*1 takes part in the cardiac endothelial cell dysfunction induced by myofibroblast-derived exosomes. Actually, *FN1* has been used as a myocardial fibrosis marker in research [37, 38]. The gene *ACTB,* encoding one of six different actin proteins, which are involved in structure, integrity, cell motility, and intercellular signaling, was enriched in the viral myocarditis pathway. Viral myocarditis is the result of direct damage to the myocardium by virus and indirect damage to the myocardium by immune response. These damage leads to edema and necrosis of the myocardial cell and proliferation and fibrosis of interstitial cell [39]. Thus, these genes may play a role in the regulation of myocardial fibrosis and then increase the risk of arrhythmia.

Recent studies gave increasing suggestion of the association of immune-related diseases (IRD) and AF. A nationwide population-based study with 37,696 patients with IBD showed that patients with IBD got a 36% (95% confidence interval = 20%-54%) higher risk of AF than controls [40]. Similarly, patients with systemic lupus erythematosus (SLE) have also been reported to get a higher risk of AF compared to controls (Hazard ratio = 2.84, 95% CI = 2.50-3.23) [41]. When it comes to the systemic sclerosis (SSc), they may get a higher risk of AF at 1.75 times of the controls (95% CI = 1.51-2.04), what is more, SSc could affect the heart and then lead to myocardial fibrosis, which would promote the formation of AF [42, 43]. Rheumatoid arthritis (RA), another autoimmune disease, has been suggested to increase the risk of AF [44]. In an early study, researchers reported that systemic inflammatory can lead to epicardial adipose tissue expansion and inflammation, and then cause the enlarger, fibrotic, and noncompliant of the left atrium, finally results in AF [45]. IL-6 is a crucial prerequisite for fibrosis of cardiac myocytes, when it causes the decrease expression of Cx40 and Cx43, it is strongly correlated with the high expression of collagen fibrin I and collagen fibrin III via the pSTAT3 pathway [46]. The expression of IL-6 increases in several IRDs, and it may be the reason that it could be suggested as a biomarker of AF [47–49]. In this study, we found that the hub gene *C3* was enriched in SLE, and *CXCL12* was enriched in rheumatoid arthritis. The expression of these genes may affect the activation of the autoimmune system, and then promotes the remodeling of the atrium, which leads to AF.

Targeted drugs of the hub genes were also identified in this study. The complement system is an important part of the human immune system and is involved in the inflammatory response. Pegcetacopla, a PEGylated peptide targeting C3, is an inhibitor of hemolysis used in clinics. It can improve hemoglobin, clinical outcome, and hematologic outcome via effect control of intravascular hemolysis as well as extravascular hemolysis [50]. C3 has also been reported associated with age and hypertension, which is known as the risk of AF. In addition, dipeptidyl peptidase III can prevent heart from inflammatory cell infiltration and fibrosis via cleavage of a peptide that is a part of C3 [51, 52]. However, little is known of the use of pegcetacopla in the treatment of heart disease. Carvedilol is one of the calcium channel blockers, and it could alter circulating miR-1 and miR-214, which are suggested in the processes of myocyte hypertrophy and apoptosis and release myocardial fibrosis [53, 54]. In addition, it can provide prevention of chemotherapy-related cardiotoxicity [55]. Thus, carvedilol may be an ideal antifibrosis target drug. Vitreomacular adhesion (VMA) is an eye disease, and it always leads to visual impairment, even loss of vision when it gets worse with vitreomacular traction (VMT). Pharmacological vitreolysis was an alternative treatment for VMA. Recently, a new drug named ocriplasmin, a recombinant DNA molecule based on autologous plasmin, was developed to catalyze the breakdown of the bond of laminin and fibronectin, maintaining vitreous adhesion [56]. The blood clots caused by AF are usually venous thrombosis, and fibrinolysis enzymes and clotting factor inhibitors are common treatments. However, whether ocriplasmin would be an effect component of anticoagulant or thrombolytic therapy still needs further researches to detect.

With the progress of technology and the continuous evolution of algorithms, our understanding of complex diseases is further deepened. Several related works provide powerful boost to medical research. Su et al. [57] reported a framework using horizontal and vertical multiverse optimization, providing an effective segmentation method for diagnosing Coronavirus Disease 2019 (COVID-19). Similarly, Qi et al. [58] reported a directional mutation and crossover boosted ant colony optimization for diagnosing COVID-19. In addition, saliency detection network with neutrosophic enhancement have been reported to be an effective approach to colorectal polyp region extraction [59]. Bioinformatics has developed rapidly in the last decade, and it was not only owing to the development of sequencing technology but also to the update of algorithms. No matter what the specific mechanism is, at present, the cause of AF is basically considered as cardiac fibrosis induced by atrial remodel. In the current study, we detected the potential mechanism of *TGFBI* and its coexpression genes of AF and found that they may induce cardiac fibrosis via several pathways. The identified hub genes may be potential targets for the interference of AF.

Based on our finding, our future work was designed as follows. Firstly, we aim to cooperate with surgeons, collecting several LAAs of AF patients, and verify the correlation of the expression levels of *TGFBI* and the identified hub genes via reverse transcription-polymerase chain reaction (RT-qPCR). Secondly, measuring the expression of proteins in the downstream of the pathway they enriched in via knocking down or overexpressing these genes to verify their function. Thirdly, after the intervention of the extracted cardiomyocytes from the LAAs with the predicted targeted drugs, the effect on the expression of these genes and proteins was evaluated by RT-qPCR and western blot. In addition, we aimed to construct an AF animal model with pacemaker via persistent rapid atrial stimulation to repeat the experiment. Finally, several representative computational intelligence algorithms, like monarch butterfly optimization (MBO) [60], earthworm optimization algorithm (EWA) [61], elephant herding optimization (EHO) [62], slime mould algorithm (SMA) [63], hunger games search (HGS) [64], RUNge Kutta optimizer (RUN) [65], colony predation algorithm (CPA) [66], and Harris hawks optimization (HHO) [67, 68] could be used to optimized our analysis.

## 5. Conclusions

In the current study, we used *TGFBI* and its coexpression genes to identify the potential molecular mechanisms of AF. These findings may help elucidate the functions of these genes in AF and provide a target of AF management. However, there were several limitations of the current study. First, the sample-size is not large as other randomized controlled study. Second, the results of this study were mainly based on bioinformatic analysis, and further experiments are needed to confirm both in vivo and in vitro. Finally, several potential factors that participant in the formation of AF may not be included. Fortunately, with the development of algorithms, several representative computational intelligence algorithms like MBO, EWA, EHO, SMA, HGS, RUN, CPA, and HHO may be used to solve the problems. In the future, the results of our study will be further verified by more optimized algorithms, expanded samples, and experiments both in vivo and in vitro.

## Figures and Tables

**Table 1:** Pathways enriched by *TGFBI* and its coexpression genes in AF patients.

Term	Count	*P* value	Enriched genes
hsa05332: Graft versus host disease	13	9.14E-08	HLA-DQB1/HLA-A/HLA-C/HLA-B/HLA-DMB/HLA-E/HLA-DMA/HLA-G/HLA-F/HLA-DPA1/HLA-DPB1/CD28/HLA-DRA
hsa04612: Antigen processing and presentation	19	1.13E-07	HLA-DQB1/PDIA3/KIR2DS5/HLA-A/HLA-C/HLA-B/KIR2DS3/HLA-DMB/HLA-E/HLA-DMA/RFXANK/CD74/HLA-G/HLA-F/CD4/HLA-DPA1/HLA-DPB1/KIR2DL3/HLA-DRA
hsa05150: *Staphylococcus aureus* infection	16	1.37E-07	HLA-DQB1/C3AR1/C3/C1R/ITGB2/C1S/HLA-DMB/HLA-DMA/C1QA/C1QB/FCGR2B/HLA-DPA1/FCGR2A/CFI/HLA-DPB1/HLA-DRA
hsa04514: Cell adhesion molecules (CAMs)	26	2.13E-07	CLDN8/CLDN16/HLA-DQB1/ITGB2/HLA-DMB/HLA-DMA/CDH5/CLDN14/SDC3/VCAM1/CD4/HLA-DPB1/CD28/ICAM2/HLA-A/CD99/HLA-C/CLDN20/HLA-B/HLA-E/HLA-G/HLA-F/NCAM2/VCAN/HLA-DPA1/HLA-DRA
hsa04940: Type I diabetes mellitus	14	2.35E-07	HLA-DQB1/HLA-A/HLA-C/HLA-B/HLA-DMB/HLA-E/HLA-DMA/HLA-G/HLA-F/CPE/HLA-DPA1/HLA-DPB1/CD28/HLA-DRA
hsa05330: Allograft rejection	13	3.89E-07	HLA-DQB1/HLA-A/HLA-C/HLA-B/HLA-DMB/HLA-E/HLA-DMA/HLA-G/HLA-F/HLA-DPA1/HLA-DPB1/CD28/HLA-DRA
hsa05320: Autoimmune thyroid disease	15	5.51E-07	HLA-DQB1/HLA-A/HLA-C/HLA-B/HLA-DMB/HLA-E/HLA-DMA/HLA-G/HLA-F/IFNA4/HLA-DPA1/IFNA14/HLA-DPB1/HLA-DRA/CD28
hsa04145: Phagosome	26	6.30E-07	HLA-DQB1/C3/C1R/ITGB2/HLA-DMB/HLA-DMA/STX12/HLA-DPB1/THBS2/TUBA1B/ACTB/MRC1/NCF2/HLA-A/COLEC12/HLA-C/HLA-B/HLA-E/HLA-G/HLA-F/FCGR2B/CD209/HLA-DPA1/FCGR2A/CD14/HLA-DRA
hsa05416: Viral myocarditis	15	1.85E-06	ACTB/HLA-DQB1/HLA-A/ITGB2/HLA-C/HLA-B/HLA-DMB/HLA-E/HLA-DMA/HLA-G/HLA-F/HLA-DPA1/HLA-DPB1/HLA-DRA/CD28
hsa04512: ECM-receptor interaction	17	1.93E-05	COL4A2/COL4A1/COL3A1/ITGA1/COL5A2/VWF/LAMA4/LAMA3/GP6/CD44/COL6A5/COL6A3/COL1A2/COL6A1/LAMB1/THBS2/FN1
hsa04610: Complement and coagulation cascades	14	8.99E-05	F13B/C1QA/C7/VWF/C3AR1/C1QB/THBD/SERPINF2/C3/TFPI/SERPING1/C1R/CFI/C1S
hsa05152: Tuberculosis	23	3.06E-04	MRC1/HLA-DQB1/SPHK2/C3/CEBPG/TIRAP/ITGB2/HLA-DMB/HLA-DMA/RFXANK/CD74/TNFRSF1A/FCGR2B/CD209/IFNA4/FCER1G/HLA-DPA1/IFNA14/FCGR2A/PPP3CA/HLA-DPB1/CD14/HLA-DRA
hsa05133: Pertussis	13	8.02E-04	C1QA/C1QB/GNAI3/C3/IRF1/TIRAP/PYCARD/ITGB2/SERPING1/C1R/C1S/CASP1/CD14
hsa05166: HTLV-I infection	28	8.39E-04	HLA-DQB1/WNT16/NRP1/ADCY8/ITGB2/HLA-DMB/HLA-DMA/VCAM1/TNFRSF1A/CDKN2A/PPP3CA/HLA-DPB1/EGR1/FZD9/IL2RB/LTBR/HLA-A/HLA-C/HLA-B/HLA-E/FZD4/HLA-G/HLA-F/ETS2/PDGFRA/PDGFRB/HLA-DPA1/HLA-DRA
hsa04672: Intestinal immune network for IgA production	10	9.65E-04	HLA-DQB1/LTBR/HLA-DPA1/TNFSF13/HLA-DPB1/HLA-DMB/HLA-DMA/CXCL12/HLA-DRA/CD28
hsa04974: Protein digestion and absorption	14	1.07E-03	COL4A2/COL14A1/COL4A1/COL7A1/COL6A5/COL3A1/COL6A3/PRCP/COL1A2/COL15A1/COL6A1/CPB1/COL5A2/SLC7A7
hsa04670: Leukocyte transendothelial migration	16	1.68E-03	ACTB/CLDN16/CLDN8/MYL7/GNAI3/NCF2/CD99/ITGB2/CLDN20/MMP2/CXCL12/CLDN14/CDH5/VCAM1/RAP1B/MSN
hsa04151: PI3K-Akt signaling pathway	33	2.83E-03	FGFR1/FGFR4/COL3A1/GNG11/LPAR1/G6PC2/COL6A5/IL4R/IFNA4/COL6A3/CREB3L2/COL6A1/LAMB1/THBS2/CSF1R/FN1/IL2RB/COL4A2/COL4A1/ITGA1/FGF21/FGF20/COL5A2/VWF/GNGT1/LAMA4/LAMA3/GNB1/LPAR6/COL1A2/PDGFRA/PDGFRB/IFNA14
hsa04810: Regulation of actin cytoskeleton	23	3.00E-03	ACTB/FGFR1/MYL7/FGFR4/ARHGEF7/ITGA1/IQGAP2/ITGB2/ARPC5/FGF21/FGF20/IQGAP1/ARPC1B/PAK3/ARPC2/TIAM1/SCIN/PDGFRA/PDGFRB/CYFIP1/TMSB4X/MSN/FN1
hsa05322: Systemic lupus erythematosus	17	3.01E-03	HLA-DQB1/C7/HIST1H4L/C3/GRIN2A/C1R/C1S/H2AFJ/HLA-DMB/HLA-DMA/C1QA/C1QB/HLA-DPA1/FCGR2A/HLA-DPB1/HLA-DRA/CD28
hsa05200: Pathways in cancer	36	3.61E-03	DCC/FGFR1/WNT16/GNAI3/ADCY8/GNG11/LPAR1/MMP2/CXCL12/CDKN2A/LAMB1/TRAF5/TRAF4/CSF1R/FN1/FZD9/BMP4/PTGER2/COL4A2/COL4A1/PTGER4/EPAS1/LEF1/FGF21/FGF20/MECOM/FZD4/GNGT1/LAMA4/LAMA3/GNAQ/GNB1/LPAR6/RASSF1/PDGFRA/PDGFRB
hsa04510: Focal adhesion	22	5.09E-03	ACTB/MYL7/COL4A2/COL4A1/COL3A1/ITGA1/FLNB/COL5A2/VWF/LAMA4/LAMA3/PAK3/COL6A5/COL6A3/COL1A2/PDGFRA/COL6A1/PDGFRB/RAP1B/LAMB1/THBS2/FN1
hsa05310: Asthma	7	5.44E-03	HLA-DQB1/FCER1G/HLA-DPA1/HLA-DPB1/HLA-DMB/HLA-DMA/HLA-DRA
hsa00590: Arachidonic acid metabolism	10	6.18E-03	AKR1C3/GGT5/PLA2G4A/PTGIS/ALOX12B/GGT1/ALOX5/CBR3/LTC4S/PLA2G2F
hsa04611: Platelet activation	15	1.30E-02	ACTB/GNAI3/ADCY8/COL3A1/COL5A2/VWF/PLA2G4A/GP6/GNAQ/VAMP8/COL1A2/FCER1G/RAP1B/FCGR2A/LCP2
hsa05140: Leishmaniasis	10	1.63E-02	HLA-DQB1/NCF2/C3/HLA-DPA1/ITGB2/FCGR2A/HLA-DPB1/HLA-DMB/HLA-DMA/HLA-DRA
hsa04666: Fc gamma R-mediated phagocytosis	11	1.76E-02	ARPC1B/FCGR2B/SPHK2/ARPC2/SCIN/ASAP2/MARCKS/FCGR2A/INPP5D/ARPC5/PLPP3
hsa05169: Epstein-Barr virus infection	14	1.77E-02	HLA-DQB1/CD44/FCER2/VIM/HLA-A/HLA-C/HLA-DPA1/HLA-B/HLA-DPB1/HLA-E/TRAF5/HLA-G/HLA-DRA/HLA-F
hsa05323: Rheumatoid arthritis	11	2.37E-02	HLA-DQB1/CTSK/HLA-DPA1/ITGB2/TNFSF13/HLA-DPB1/HLA-DMB/HLA-DMA/CXCL12/HLA-DRA/CD28
hsa05321: Inflammatory bowel disease (IBD)	9	2.47E-02	MAF/HLA-DQB1/IL4R/HLA-DPA1/HLA-DPB1/HLA-DMB/IL21/HLA-DMA/HLA-DRA
hsa05168: Herpes simplex infection	18	2.57E-02	HLA-DQB1/C3/HLA-A/HLA-C/HLA-B/HLA-DMB/HLA-E/HLA-DMA/HLA-G/CD74/HLA-F/TNFRSF1A/IFNA4/HLA-DPA1/IFNA14/HLA-DPB1/TRAF5/HLA-DRA
hsa04380: Osteoclast differentiation	14	3.00E-02	NOX3/NCF2/NOX1/TNFRSF1A/CYBB/CTSK/LILRA1/FCGR2B/OSCAR/FCGR2A/PPP3CA/LCP2/CSF1R/TYROBP
hsa05146: Amoebiasis	12	3.31E-02	COL4A2/LAMA4/LAMA3/COL4A1/GNAQ/COL3A1/COL1A2/ITGB2/LAMB1/COL5A2/CD14/FN1
hsa04650: Natural killer cell mediated cytotoxicity	13	3.84E-02	CD244/TNFSF10/KIR2DS5/ICAM2/IFNA4/FCER1G/IFNA14/ITGB2/PPP3CA/KIR2DS3/KIR2DL3/LCP2/TYROBP
hsa04390: Hippo signaling pathway	15	4.12E-02	ACTB/BMP4/FZD9/WNT16/LEF1/ITGB2/WWTR1/FZD4/FRMD1/AFP/CRB1/ID2/ID1/RASSF1/TEAD4
hsa05145: Toxoplasmosis	12	4.18E-02	HLA-DQB1/TNFRSF1A/LAMA4/LAMA3/GNAI3/HLA-DPA1/ALOX5/HLA-DPB1/HLA-DMB/LAMB1/HLA-DMA/HLA-DRA
hsa04015: Rap1 signaling pathway	19	4.43E-02	ACTB/FGFR1/FGFR4/GNAI3/ADCY8/GRIN2A/ITGB2/LPAR1/FGF21/FGF20/GNAQ/ID1/TIAM1/PDGFRA/RAPGEF5/PDGFRB/RAP1B/LCP2/CSF1R
hsa04014: Ras signaling pathway	20	4.66E-02	FGFR1/FGFR4/GRIN2A/GNG11/FGF21/FGF20/RGL1/GNGT1/PLA2G4A/PAK3/GNB1/TIAM1/RASSF1/ETS2/PDGFRA/RAPGEF5/PDGFRB/RAP1B/PLA2G2F/CSF1R
hsa04360: Axon guidance	13	4.97E-02	DCC/NRP1/GNAI3/PLXNA1/EFNB2/CXCL12/EPHB1/SLIT2/SEMA5A/ROBO1/PAK3/UNC5D/PPP3CA

**Table 2:** Validation of the correlations between the hub genes and *TGFBI*.

Gene	Degree rank	GSE115574		GSE79768	
		cor	*P*	cor	*P*
*CXCL12* ^∗^	6	0.915	4.61E-06	0.839	1.82E-02
*C3* ^∗^	6	0.733	2.87E-03	0.857	1.38E-02
*FN1* ^∗^	1	0.807	4.86E-04	0.813	2.60E-02
*COL1A2* ^∗^	6	0.821	3.18E-04	0.801	3.04E-02
*ACTB* ^∗^	4	0.770	1.28E-03	0.842	1.74E-02
*VCAM1* ^∗^	6	0.768	1.33E-03	0.757	4.87E-02
*MMP2* ^∗^	3	0.922	2.73E-06	0.917	3.68E-03
*VWF*	10	0.770	1.29E-03	0.625	1.34E-01
*BMP4*	5	0.746	2.18E-03	0.527	2.24E-01
*CD44*	2	0.856	9.24E-05	0.619	1.38E-01

^∗^ *P* < 0.05in both dataset. cor: Pearson correlation value.

**Table 3:** Target drug of the hub genes.

Gene	Drug ID	Name	Status	Actions
*C3*	DB00028	Human immunoglobulin G	Approved	Binder
	DB14533	Zinc chloride	Approved	Inhibitor, ligand
	DB14548	Zinc sulfate	Approved	Inhibitor, ligand
	DB16694	Pegcetacoplan	Approved	Binder, regulator

*FN1*	DB08888	Ocriplasmin	Approved	Cleavage
	DB14533	Zinc chloride	Approved	Modulator, ligand
	DB14548	Zinc sulfate	Approved	Modulator, ligand

*VCAM1*	DB01136	Carvedilol	Approved	Inhibitor
	DB11338	Clove oil	Approved	Antagonist

## Data Availability

The datasets used and/or analyzed during the current study are available from the Gene Expression Omnibus repository (https://www.ncbi.nlm.nih.gov/geo/query/acc.cgi? GEO accession: GSE115574 and GSE79768).

## Abbreviations

AF: Atrial fibrillation
*TGFBI*: Transforming growth factor beta induced
GEO: Gene Expression Omnibus
GO: Gene Ontology
KEGG: Kyoto Encyclopedia of Genes and Genomes pathway enrichment analyses
PPI: Protein-protein interaction
BP: Biological processes
CC: Cellular components
MF: Molecular functions
IBD: Inflammatory bowel disease
SLE: Systemic lupus erythematosus
IRD: Immune-related diseases
SSc: Systemic sclerosis
VMA: Vitreomacular adhesion.

## Supplementary Materials

Supplementary Materials Table S1: GO annotation of *TGFBI* and coexpression genes enriched in AF. Table S2: GO annotation and KEGG pathway *TGFBI* and coexpression genes enriched in cluster 1. Figure S1: the clusters constructed from the PPI-network.
