# Supplementary material for: Identification of the Potential Molecular Mechanism of TGFBI Gene in Persistent Atrial Fibrillation
Source: Comput Math Methods Med. 2022 Nov 8;2022:1643674. doi: 10.1155/2022/1643674 (PMC9666036; doi:10.1155/2022/1643674)
Supplement: Supplementary Materials — Table S1: GO annotation of TGFBI and coexpression genes enriched in AF. Table S2: GO annotation and KEGG pathway TGFBI and coexpression genes enriched in cluster 1. Figure S1: the clusters constructed from the PPI-network. [file 1643674.f1.zip › Table S1. GO annotation of TGFBI and co-expression genes enriched in AF.pdf]

**Table S1. GO annotation of TGFBI and co-expression genes enriched in AF**

| Category | Term | Count | % | PValue | Genes |
|---|---|---|---|---|---|
| BP | GO:0030198~extracellular matrix organization | 34 | 0.027 | 3.55E-10 | LUM/COL3A1/ITGB2/DCN/VCAM1/DSPP/CD44/COL7A1/CRISPLD2/TGFBI/COL6A3/COL6A1/LOX/LAMB1/LOXL1/FN1/RECK/COL4A2/COL4A1/ICAM2/FBN1/NOX1/ITGA1/SPARC/ECM2/COL5A2/VWF/LAMA4/FBLN1/COL14A1/LAMA3/FBLN5/COL1A2/VCAN |
| BP | GO:0030199~collagen fibril organization | 12 | 0.009 | 1.58E-06 | FMOD/COL14A1/CYP1B1/SERPINF2/LUM/COL3A1/COL1A2/LOX/SERPINH1/COL5A2/ANXA2/DPT |
| BP | GO:0019882~antigen processing and presentation | 14 | 0.011 | 1.64E-06 | HLA-DQB1/HLA-A/FCGRT/HLA-C/HLA-B/HLA-DMB/HLA-E/HLA-G/CD74/RAB32/CD209/HLA-DPA1/HLA-DPB1/HLA-DRA |
| BP | GO:0030574~collagen catabolic process | 15 | 0.012 | 1.79E-06 | KLK6/COL4A2/PEPD/COL4A1/COL3A1/COL15A1/COL5A2/MMP2/MMP13/CTSK/COL7A1/COL6A5/COL6A3/COL1A2/COL6A1 |
| BP | GO:0045087~innate immune response | 44 | 0.034 | 5.82E-06 | FRK/CD244/ATG12/ZC3HAV1/S100A7/LY86/CLU/TIRAP/C1R/C1S/TRIM10/NLRC4/CASP4/TKFC/RNASE7/IFNA4/PYCARD/FCER1G/ITCH/CFI/TYROBP/CSF1R/NCF2/BST2/KIR2DS5/ANXA1/SLAMF6/HLA-C/SERPING1/COLEC12/HLA-B/IFI16/HLA-E/SIGLEC15/GZMM/C1QA/C1QB/CYBB/APOL1/CD209/IFNA14/TREM1/PRDM1/CD14 |
| BP | GO:0060337~type I interferon signaling pathway | 14 | 0.011 | 9.94E-06 | EGR1/BST2/IFITM2/IFITM3/HLA-A/HLA-C/HLA-B/HLA-E/HLA-G/HLA-F/IFI27/IFNA4/IRF1/IFNA14 |
| BP | GO:0007155~cell adhesion | 45 | 0.035 | 1.33E-05 | CYP1B1/CASK/ITGB2/CXCL12/CDH5/ITGBL1/SEMA5A/ISLR/VCAM1/CD9/DSPP/CD44/COL7A1/COL6A5/ROBO1/TGFBI/COL6A3/COL6A1/CD4/LAMB1/THBS2/DPT/SPON1/FN1/TYRO3/SVEP1/ICAM2/EFNB2/COL15A1/CD99/CHST4/AJAP1/TPBG/PRPH2/VWF/NCAM2/LAMA4/OMD/LAMA3/SIGLEC6/VCAN/ADAM12/THEMIS2/AOC3/CDH11 |
| BP | GO:0002480~antigen processing and presentation of exogenous peptide antigen via MHC class I, TAP-independent | 6 | 0.005 | 2.85E-05 | HLA-A/HLA-C/HLA-B/HLA-E/HLA-G/HLA-F |
| BP | GO:0060333~interferon-gamma | 14 | 0.011 | 3.21E-05 | HLA-DQB1/HLA-A/HLA-C/HLA-B/HLA-E/TRIM22/HLA-G/HLA-F/VCAM1/CD44/IRF1/H |

| | | | | | |
|---|---|---|---|---|---|
| | -mediated signaling pathway | | | | LA-DPA1/HLA-DPB1/HLA-DRA |
| BP | GO:0050776~regulation of immune response | 23 | 0.018 | 5.29E-05 | C3/LAIR2/ICAM2/COL3A1/HLA-A/SLAMF6/CD1B/HLA-C/ITGB2/HLA-B/HLA-E/HLA-G/HLA-F/VCAM1/LILRA1/FCGR2B/CLEC2B/OSCAR/COL1A2/IRF1/TREM1/KIR2DL3/TYROBP |
| BP | GO:0006955~immune response | 39 | 0.030 | 1.80E-04 | HLA-DQB1/C7/LST1/IFITM2/CYP11B1/C3/IFITM3/TNFSF13/C1R/HLA-DMB/HLA-DMA/CXCL12/CD74/MBP/TNFRSF1A/IL4R/CD4/HLA-DPB1/LTBR/PTGER4/KIR2DS5/CEBPG/HLA-A/HLA-C/CHST4/HLA-B/GEM/IL21/CCL16/HLA-E/TRIM22/HLA-F/TNFSF10/FCGR2B/LAX1/HLA-DPA1/KIR2DL3/LCP2/HLA-DRA |
| BP | GO:0035987~endodermal cell differentiation | 8 | 0.006 | 2.34E-04 | COL4A2/LAMA3/COL7A1/COL6A1/ITGB2/LAMB1/MMP2/FN1 |
| BP | GO:0002474~antigen processing and presentation of peptide antigen via MHC class I | 8 | 0.006 | 4.72E-04 | HLA-DQB1/PDIA3/HLA-A/HLA-C/HLA-B/HLA-E/HLA-G/HLA-F |
| BP | GO:0071456~cellular response to hypoxia | 14 | 0.011 | 7.31E-04 | CLCA1/EPAS1/SUV39H1/NPEPPS/AQP1/ZFP36L1/GNGT1/PTGIS/GNB1/HMOX1/BNIP3L/PTN/NDRG1/TWIST1 |
| BP | GO:0050900~leukocyte migration | 16 | 0.012 | 8.33E-04 | C3AR1/CD244/ITGB2/MYH9/CD74/SLC7A7/THBD/GP6/CD44/COL1A2/FCER1G/MSN/INPP5D/TREM1/FN1/NKX2-3 |
| BP | GO:0007165~signal transduction | 81 | 0.063 | 8.89E-04 | NRP1/PDIA3/ADCY8/IGFBP6/TIRAP/IQGAP2/TNFSF13/CD53/TNFSF18/CXCL12/ARL2BP/IQGAP1/ARHGAP6/ANK1/CHRNA9/IL4R/PITPNC1/UNC5D/CAP1/CASP1/TYRO3/LTBR/SPARCL1/ARHGEF7/CLIC2/CLIC1/GEM/INHA/FGF21/ALK/IL21/FGF20/FLNB/GABRR2/THBD/PRDM4/GNB1/PDGFRB/AMFR/SH3GL2/CD244/C3/GNG11/ELK3/SP110/GPR143/CD74/TNFRSF1A/NPHP4/PYCARD/CD4/INPP5D/TRAF5/TRAF4/TYROBP/CSF1R/MRC1/IL2RB/EPAS1/KIR2DS5/LGALS1/GABRA5/NOX1/CACNA1I/S100A11/ANXA1/COL15A1/SPARC/ECM1/ANXA4/SH3BP5/GNGT1/APOL3/RPS6KA6/P2RX4/TNFSF10/ZNF217/FCGR2B/CHRNB3/CHRNA10/PLCXD2 |
| BP | GO:0002504~antigen | 6 | 0.005 | 1.01E-03 | HLA-DQB1/HLA-DPA1/HLA-DPB1/HLA-DMB/HLA-DMA/HLA-DRA |

| | | | | | |
|---|---|---|---|---|---|
| | processing and presentation of peptide or polysaccharide antigen via MHC class II | | | | |
| BP | GO:0001525~angiogenesis | 23 | 0.018 | 1.28E-03 | PTPRB/FGFR1/COL4A2/NRP1/CYP1B1/EPAS1/S100A7/NOX1/COL15A1/JAG1/ELK3/MYH9/ECM1/MMP2/EPHB1/ANXA2/MMRN2/MEOX2/ID1/PLXDC1/HMOX1/TGFBI/FN1 |
| BP | GO:0071230~cellular response to amino acid stimulus | 9 | 0.007 | 1.74E-03 | COL4A1/GLRA1/COL3A1/NTRK2/COL1A2/DNMT1/COL6A1/MMP2/COL5A2 |
| BP | GO:0010951~negative regulation of endopeptidase activity | 15 | 0.012 | 2.20E-03 | BST2/C3/SERPING1/SERPINH1/TIMP1/WFDC8/SERPINE2/CARD16/SERPINF1/COL7A1/SERPINF2/COL6A3/TFPI/ITIH5/CRIM1 |
| BP | GO:0001501~skeletal system development | 16 | 0.012 | 2.69E-03 | FGFR1/COL3A1/FBN1/INHA/COL5A2/PTHLH/DSPP/GNAQ/ETS2/TEAD4/COL1A2/VCAN/PAPSS1/PAPSS2/SERP1/CDH11 |
| BP | GO:0006928~movement of cell or subcellular component | 12 | 0.009 | 2.90E-03 | ACTB/CD9/ACTR2/ARPC1B/ARPC2/CALD1/VIM/CHST4/MSN/AMFR/ARPC5/ARHGDIB |
| BP | GO:0043065~positive regulation of apoptotic process | 27 | 0.021 | 3.13E-03 | ING3/PNMA2/CYP1B1/CLU/LPAR1/NLRC4/CDKN2A/TIAM1/HMOX1/MAP3K9/PYCARD/PTN/INPP5D/FZD9/BMP4/ARHGEF7/GRIN2A/SLIT2/TNFSF10/SFRP4/BNIP3L/SCIN/PDGFRB/NEUROD1/ID3/CLIP3/DUSP6 |
| BP | GO:0006954~inflammatory response | 32 | 0.025 | 3.25E-03 | C3AR1/NDST1/AIF1/C3/LY86/TACR1/TIRAP/ITGB2/CXCL12/TNFRSF1A/NLRC4/CASP4/CXCR6/PYCARD/ITCH/CSF1R/PTGER2/LTBR/LIPA/NOX1/ANXA1/IL25/CHST4/IFI16/CCL16/ECM1/GGT5/APOL3/CYBB/THEMIS2/CD14/AOC3 |
| BP | GO:0007596~blood coagulation | 19 | 0.015 | 3.72E-03 | RAD51B/HPS5/F13B/P2RX4/VWF/GATA2/THBD/GP6/GNAQ/SERPINE2/IFNA4/TFPI/COL1A2/VKORC1/IRF1/LNPK/FCER1G/IFNA14/PAPSS2 |
| BP | GO:0045766~positive regulation of angiogenesis | 14 | 0.011 | 3.81E-03 | C3AR1/CYP1B1/MTDH/C3/ITGB2/AQP1/ECM1/SEMA5A/GATA2/CYBB/PTGIS/HMOX1/DDAH1/TWIST1 |
| BP | GO:0060326~cell chemotaxis | 10 | 0.008 | 3.98E-03 | SEMA5A/VCAM1/C3AR1/PDGFRA/PDGFRB/LEF1/LPAR1/CCL16/CXCL12/EPHB1 |

| | | | | | |
|---|---|---|---|---|---|
| BP | GO:0002576~platelet degranulation | 13 | 0.010 | 4.18E-03 | ISLR/CD9/VWF/POTEKP/SERPINF2/CLU/SERPING1/TMSB4X/SPARC/ECM1/SRGN/FN1/TIMP1 |
| BP | GO:0007275~multicellular organism development | 40 | 0.031 | 4.89E-03 | WNT16/LMO2/PLXNA1/LTBP4/PRTG/PAX6/JAG1/PCOLCE/HEMGN/TCP11/DAB2/OLFML3/DSPP/DND1/MN1/CSF1R/ARHGDIB/FZD9/LTBR/BST2/LMX1B/PMCH/RYBP/PIM1/ACRV1/CSRP2/FZD4/THEG/TNP2/CYLC1/HOXB2/ID2/SERPINF1/MEOX2/EBF2/SFRP4/TGIF1/VCAN/ID3/ADRA1D |
| BP | GO:0006691~leukotriene metabolic process | 5 | 0.004 | 4.89E-03 | GGT5/CYP4A11/GGT1/ALOX5/LTC4S |
| BP | GO:0001503~ossification | 11 | 0.009 | 5.22E-03 | FZD9/DSPP/CHRDL1/MGP/LRRC17/SPARC/RUNX2/ECM1/COL5A2/CDH11/TWIST1 |
| BP | GO:0060124~positive regulation of growth hormone secretion | 4 | 0.003 | 5.31E-03 | GHRH/ARHGEF7/GHRHR/SERP1 |
| BP | GO:0002486~antigen processing and presentation of endogenous peptide antigen via MHC class I via ER pathway, TAP-independent | 3 | 0.002 | 6.83E-03 | HLA-A/HLA-C/HLA-B |
| BP | GO:0072277~metanephric glomerular capillary formation | 3 | 0.002 | 6.83E-03 | TCF21/PDGFRA/PDGFRB |
| BP | GO:0048469~cell maturation | 7 | 0.005 | 7.18E-03 | FGFR1/GATA2/ID2/EPAS1/IL21/RUNX2/GHRHR |
| BP | GO:0007166~cell surface receptor signaling pathway | 24 | 0.019 | 7.67E-03 | GPR19/TACR1/ADGRF5/ANXA1/IL25/TIRAP/OXTR/GEM/INHA/CD53/GPR88/PRPH2/GHRHR/ADGRG4/CD9/TNFSF10/LILRA1/NPPB/CD4/IL13RA1/ADGRA2/CD14/ADGRL4/CD28 |
| BP | GO:0032330~regulation of chondrocyte differentiation | 4 | 0.003 | 7.68E-03 | MAF/PTHLH/SCIN/LNPK |
| BP | GO:0071404~cellular response | 4 | 0.003 | 7.68E-03 | CD9/FCER1G/ITGB2/FGF21 |

| | | | | | |
|---|---|---|---|---|---|
| | to low-density lipoprotein particle stimulus | | | | |
| BP | GO:0061314~Notch signaling involved in heart development | 4 | 0.003 | 7.68E-03 | HEY2/JAG1/GALNT11/SNAI1 |
| BP | GO:0090197~positive regulation of chemokine secretion | 4 | 0.003 | 7.68E-03 | IL4R/PYCARD/IL33/CSF1R |
| BP | GO:0042102~positive regulation of T cell proliferation | 9 | 0.007 | 8.14E-03 | VCAM1/AIF1/ANXA1/CD4/HLA-DPA1/HLA-DPB1/HLA-DMB/IL21/CD28 |
| BP | GO:0048013~ephrin receptor signaling pathway | 11 | 0.009 | 8.66E-03 | ACTB/ACTR2/ARPC1B/TIAM1/PAK3/ARHGEF7/ARPC2/EFNB2/ARPC5/MMP2/EPHB1 |
| BP | GO:0042476~odontogenesis | 6 | 0.005 | 8.82E-03 | BMP4/COL1A2/ID3/AQP1/LAMB1/TWIST1 |
| BP | GO:0001568~blood vessel development | 7 | 0.005 | 9.38E-03 | LAMA4/SPHK2/COL1A2/PAX6/STRA6/GJA4/PLPP3 |
| BP | GO:0060291~long-term synaptic potentiation | 7 | 0.005 | 9.38E-03 | STX4/SERPINE2/SLC24A2/LRRTM2/NTRK2/GRIN2A/PTN |
| BP | GO:0035904~aorta development | 5 | 0.004 | 9.77E-03 | ADAMTS6/NDST1/PRDM1/LOX/LOXL1 |
| BP | GO:0051965~positive regulation of synapse assembly | 9 | 0.007 | 9.89E-03 | BDNF/LRRTM2/LRTM1/NTRK2/LRTM2/OXTR/THBS2/TPBG/EPHB1 |
| BP | GO:0016525~negative regulation of angiogenesis | 9 | 0.007 | 9.89E-03 | COL4A2/SERPINF1/SULF1/NPPB/PTN/DCN/SPARC/TCF4/THBS2 |
| BP | GO:0007189~adenylate cyclase-activating G-protein coupled receptor signaling pathway | 8 | 0.006 | 1.00E-02 | PTHLH/PTGER2/PTGER4/GNAQ/GHRH/ADCY8/DRD5/GHRHR |

| | | | | | |
|---|---|---|---|---|---|
| BP | GO:0043086~negative regulation of catalytic activity | 10 | 0.008 | 1.02E-02 | HSPBP1/SNCB/CABP1/PTN/PRPSAP1/ANXA4/PPP1R14A/ANXA2/TIMP1/ANXA2P2 |
| BP | GO:0003184~pulmonary valve morphogenesis | 4 | 0.003 | 1.06E-02 | BMP4/HEY2/STRA6/JAG1 |
| BP | GO:0009611~response to wounding | 9 | 0.007 | 1.09E-02 | ZFP36L1/KLK6/VWF/NRP1/GRIN2A/PAX6/ID3/FABP5/FN1 |
| BP | GO:0002479~antigen processing and presentation of exogenous peptide antigen via MHC class I, TAP-dependent | 9 | 0.007 | 1.09E-02 | CYBB/NCF2/HLA-A/HLA-C/HLA-B/HLA-E/HLA-G/PSMB9/HLA-F |
| BP | GO:0006915~apoptotic process | 41 | 0.032 | 1.15E-02 | ARL6IP1/DCC/IER3/LY86/FAM3B/ITGB2/RTN3/PLAGL1/ZFP36L1/NLRC4/DAB2/CASP4/CDKN2A/TIAM1/MAP3K9/PYCARD/ZNF443/UNC5D/ITCH/INPP5D/DAP/CASP1/TRAF5/TRAF4/KLLN/LTBR/LGALS1/CST3/PIM1/RYBP/ZSWIM2/MECOM/GZMM/BFAR/TNFSF10/SULF1/IRF1/PPP1R13B/DRAM1/FAIM2/CD14 |
| BP | GO:0043123~positive regulation of I-kappaB kinase/NF-kappaB signaling | 16 | 0.012 | 1.18E-02 | S100A4/LTBR/MTDH/BST2/ZC3HAV1/LGALS1/TIRAP/LPAR1/TRIM22/ECM1/TNFRSF1A/APOL3/TNFSF10/HMOX1/CASP1/TRAF5 |
| BP | GO:0006508~proteolysis | 37 | 0.029 | 1.19E-02 | PDIA3/C3/CPQ/C1R/C1S/MMP2/PCOLCE/PRSS8/CASP4/ADAM30/CFI/CASP1/DPP7/ADAM7/TMPRSS15/CLCA1/AGBL1/PEPD/CLCA4/CAPN9/KLK1/NPEPPS/MMP13/GZMM/C1QA/GGT5/ADAMTS6/C1QB/CTSK/CTSO/TMPRSS11E/PRCP/FBXL6/ADAM12/CPB1/MST1L/RNPEP |
| BP | GO:0007267~cell-cell signaling | 22 | 0.017 | 1.23E-02 | HCN2/NRP1/BST2/EFNB2/ITGB2/CHST4/INHA/FGF21/CCL16/FGF20/GJA4/TNFSF18/SEMA5A/PTHLH/C1QA/TNFSF10/BDNF/CRB1/SIGLEC6/GHRH/LHX1/ADRA1D |
| BP | GO:0031295~T cell costimulation | 10 | 0.008 | 1.30E-02 | HLA-DQB1/BTLA/PAK3/LGALS1/EFNB2/CD4/HLA-DPA1/HLA-DPB1/HLA-DRA/CD28 |
| BP | GO:0061304~retinal blood | 3 | 0.002 | 1.32E-02 | COL4A1/CYP1B1/FZD4 |

| | | | | | |
|---|---|---|---|---|---|
| | vessel morphogenesis | | | | |
| BP | GO:0016064~immunoglobulin mediated immune response | 4 | 0.003 | 1.40E-02 | IL4R/FCER1G/INPP5D/CD74 |
| BP | GO:0060348~bone development | 7 | 0.005 | 1.51E-02 | AMER1/PTGER4/SULF1/VKORC1/SPARC/PAPSS2/PLS3 |
| BP | GO:0042060~wound healing | 10 | 0.008 | 1.52E-02 | DRD5/COL3A1/PDGFRA/PDGFRB/LOX/DCN/ELK3/SPARC/FN1/TIMP1 |
| BP | GO:0007507~heart development | 17 | 0.013 | 1.67E-02 | POU6F1/COL3A1/FBN1/OXTR/BICC1/SPARC/BORCS8/ZFP36L1/GATA2/C1ORF127/GNAQ/ROBO1/ID1/PTN/STRA6/ID3/LOX |
| BP | GO:0045429~positive regulation of nitric oxide biosynthetic process | 7 | 0.005 | 1.69E-02 | P2RX4/AIF1/CLU/ITGB2/DDAH2/DDAH1/KLF4 |
| BP | GO:0007191~adenylate cyclase-activating dopamine receptor signaling pathway | 4 | 0.003 | 1.80E-02 | DRD3/GNB1/DRD5/GPR52 |
| BP | GO:0030208~dermatan sulfate biosynthetic process | 4 | 0.003 | 1.80E-02 | CHST12/VCAN/DCN/DSE |
| BP | GO:0008284~positive regulation of cell proliferation | 34 | 0.027 | 1.94E-02 | FGFR1/FGFR4/RAD51B/DRD3/TNFSF13/POU1F1/GHRHR/TIMP1/AKR1C3/TIAM1/PTN/RAB25/RUNX2/CSF1R/FN1/SHMT2/STX4/SPHK2/EFNB2/NOX1/CST3/ROGDI/LEF1/VWCE/FGF21/IL21/FGF20/WWTR1/PTHLH/GHRH/NTRK2/PDGFRA/PDGFRB/ADRA1D |
| BP | GO:0034314~Arp2/3 complex-mediated actin nucleation | 5 | 0.004 | 2.00E-02 | ACTR2/ARPC1B/ARPC2/IQGAP2/ARPC5 |
| BP | GO:0008015~blood circulation | 7 | 0.005 | 2.08E-02 | C3AR1/HOXB2/FLI1/MEOX2/SERPING1/CXCL12/MYOF |
| BP | GO:0061156~pulmonary artery morphogenesis | 3 | 0.002 | 2.13E-02 | HEY2/STRA6/JAG1 |

| | | | | | |
|---|---|---|---|---|---|
| BP | GO:0050920~regulation of chemotaxis | 3 | 0.002 | 2.13E-02 | EFNB2/PDGFRA/ADGRA2 |
| BP | GO:0002503~peptide antigen assembly with MHC class II protein complex | 3 | 0.002 | 2.13E-02 | HLA-DMB/HLA-DMA/HLA-DRA |
| BP | GO:0021879~forebrain neuron differentiation | 3 | 0.002 | 2.13E-02 | POU3F4/LEF1/CSF1R |
| BP | GO:2000566~positive regulation of CD8-positive, alpha-beta T cell proliferation | 3 | 0.002 | 2.13E-02 | CD244/HLA-A/HLA-E |
| BP | GO:0042270~protection from natural killer cell mediated cytotoxicity | 3 | 0.002 | 2.13E-02 | HLA-A/HLA-B/HLA-E |
| BP | GO:0070373~negative regulation of ERK1 and ERK2 cascade | 8 | 0.006 | 2.16E-02 | RPS6KA6/SPRY1/FBLN1/DUSP26/PTPN2/PTPN1/KLF4/DUSP6 |
| BP | GO:0008340~determination of adult lifespan | 4 | 0.003 | 2.26E-02 | INPP5D/POU1F1/RAD54L/GHRHR |
| BP | GO:0007009~plasma membrane organization | 4 | 0.003 | 2.26E-02 | CRB1/FAT4/SPTBN1/SERP1 |
| BP | GO:0043518~negative regulation of DNA damage response, signal transduction by p53 class mediator | 4 | 0.003 | 2.26E-02 | CD44/SNAI1/CD74/TWIST1 |
| BP | GO:0043066~negative regulation of apoptotic process | 33 | 0.026 | 2.30E-02 | ARL6IP1/IER3/MTDH/AIF1/PPT1/AQP1/TNFSF18/CD74/NAA35/TIMP1/ATAD3A/DAB2/CD44/PAX7/ITCH/DDAH2/CSF1R/TWIST1/BMP4/IL2RB/SPHK2/TMBIM6/ANXA1/PI |

| | | | | | |
|---|---|---|---|---|---|
| | | | | | M1/LEF1/ANXA4/BFAR/MAP4K4/ID1/BNIP3L/PDGFRB/FAIM2/TEX11 |
| BP | GO:0070374~positive regulation of ERK1 and ERK2 cascade | 16 | 0.012 | 2.35E-02 | BMP4/FGFR4/NRP1/S100A7/TIRAP/FGF21/CCL16/FGF20/CD74/CD44/SERPINF2/PYCARD/PDGFRA/PDGFRB/RAP1B/CSF1R |
| BP | GO:0032760~positive regulation of tumor necrosis factor production | 7 | 0.005 | 2.53E-02 | CLU/TIRAP/PYCARD/FCER1G/HLA-E/CD14/TWIST1 |
| BP | GO:0043433~negative regulation of sequence-specific DNA binding transcription factor activity | 8 | 0.006 | 2.56E-02 | ID2/ID1/CEBPG/HMOX1/SFRP4/PIM1/ID3/TWIST1 |
| BP | GO:0030334~regulation of cell migration | 9 | 0.007 | 2.66E-02 | LAMA4/LAMA3/SERPINE2/PLXNA1/SRCIN1/LMO4/PAX6/DPYSL3/JAG1 |
| BP | GO:1902230~negative regulation of intrinsic apoptotic signaling pathway in response to DNA damage | 4 | 0.003 | 2.78E-02 | CLU/TMEM161A/CXCL12/SNAI1 |
| BP | GO:0042554~superoxide anion generation | 4 | 0.003 | 2.78E-02 | NOX3/CYBB/NCF2/NOX1 |
| BP | GO:0002250~adaptive immune response | 14 | 0.011 | 2.81E-02 | BTLA/CD244/BTNL8/LILRA1/LAX1/CD209/IFNA4/ANXA1/CD1B/CD4/IFNA14/PRDM1/HLA-E/TNFSF18 |
| BP | GO:0051607~defense response to virus | 15 | 0.012 | 3.01E-02 | APOBEC1/BST2/ZC3HAV1/IFITM2/IFITM3/FAM111A/IFI16/IL33/TRIM22/IFNA4/BNIP3L/PYCARD/IRF1/IFNA14/ITCH |
| BP | GO:0022617~extracellular matrix disassembly | 9 | 0.007 | 3.07E-02 | CTSK/LAMA3/CD44/FBN1/DCN/MMP13/MMP2/FN1/TIMP1 |
| BP | GO:0007159~leukocyte cell-cell | 5 | 0.004 | 3.09E-02 | APOA4/VCAM1/CD209/ITGB2/MSN |

| | | | | | |
|---|---|---|---|---|---|
| | adhesion | | | | |
| BP | GO:0019369~arachidonic acid metabolic process | 5 | 0.004 | 3.09E-02 | CYP4A11/PLA2G4A/CYP1B1/FAAH/ALOX12B |
| BP | GO:0030206~chondroitin sulfate biosynthetic process | 5 | 0.004 | 3.09E-02 | CHST12/VCAN/DCN/CHST15/DSE |
| BP | GO:0060426~lung vasculature development | 3 | 0.002 | 3.10E-02 | TCF21/ID1/STRA6 |
| BP | GO:0001765~membrane raft assembly | 3 | 0.002 | 3.10E-02 | S100A10/RFTN1/ANXA2 |
| BP | GO:0001957~intramembranous ossification | 3 | 0.002 | 3.10E-02 | CTSK/MN1/MMP2 |
| BP | GO:0002042~cell migration involved in sprouting angiogenesis | 4 | 0.003 | 3.35E-02 | NRP1/ROBO1/EFNB2/SLIT2 |
| BP | GO:0019886~antigen processing and presentation of exogenous peptide antigen via MHC class II | 10 | 0.008 | 3.44E-02 | HLA-DQB1/KIF15/FCER1G/HLA-DPA1/HLA-DPB1/HLA-DMB/HLA-DMA/SH3GL2/CD74/HLA-DRA |
| BP | GO:0035025~positive regulation of Rho protein signal transduction | 5 | 0.004 | 3.52E-02 | ROBO1/LPAR6/COL3A1/PDGFRB/LPAR1 |
| BP | GO:0001657~ureteric bud development | 6 | 0.005 | 3.55E-02 | BMP4/TCF21/FGFR1/SPRY1/LHX1/SLIT2 |
| BP | GO:0002062~chondrocyte differentiation | 6 | 0.005 | 3.91E-02 | BMP4/FGFR1/TGFBI/CREB3L2/RUNX2/NFIB |
| BP | GO:0050890~cognition | 6 | 0.005 | 3.91E-02 | PTCHD1/TMPRSS11E/CYFIP1/STRA6/CBR3/HLA-DRA |

| | | | | | |
|---|---|---|---|---|---|
| BP | GO:0035329~hippo signaling | 5 | 0.004 | 3.98E-02 | PJA2/NPHP4/FAT4/TEAD4/WWTR1 |
| BP | GO:0010575~positive regulation of vascular endothelial growth factor production | 5 | 0.004 | 3.98E-02 | C3AR1/CYP1B1/C3/SULF1/NOX1 |
| BP | GO:0090026~positive regulation of monocyte chemotaxis | 4 | 0.003 | 3.98E-02 | S100A7/AIF1/CXCL12/TNFSF18 |
| BP | GO:0090280~positive regulation of calcium ion import | 4 | 0.003 | 3.98E-02 | TRPV2/PDGFRB/CASK/CXCL12 |
| BP | GO:0030183~B cell differentiation | 8 | 0.006 | 4.03E-02 | FZD9/VCAM1/KLF6/PTPN2/CEBPG/IFNA4/IFNA14/POU1F1 |
| BP | GO:0045650~negative regulation of macrophage differentiation | 3 | 0.002 | 4.20E-02 | GATA2/PTPN2/INHA |
| BP | GO:0031340~positive regulation of vesicle fusion | 3 | 0.002 | 4.20E-02 | ANXA1/DOC2B/ANXA2 |
| BP | GO:0030252~growth hormone secretion | 3 | 0.002 | 4.20E-02 | GHRH/LTBP4/GHRHR |
| BP | GO:0043567~regulation of insulin-like growth factor receptor signaling pathway | 3 | 0.002 | 4.20E-02 | IGFBP6/POU1F1/GHRHR |
| BP | GO:0048842~positive regulation of axon extension involved in axon guidance | 3 | 0.002 | 4.20E-02 | SEMA5A/NRP1/CXCL12 |
| BP | GO:0051099~positive | 3 | 0.002 | 4.20E-02 | S100A10/CLIC2/ANXA2 |

| | | | | | |
|---|---|---|---|---|---|
| | regulation of binding | | | | |
| BP | GO:0045071~negative regulation of viral genome replication | 6 | 0.005 | 4.30E-02 | ZC3HAV1/BST2/IFITM2/IFITM3/FAM111A/IFI16 |
| BP | GO:0043392~negative regulation of DNA binding | 5 | 0.004 | 4.47E-02 | ID2/HMOX1/LEF1/IFI16/NFIB |
| BP | GO:0048146~positive regulation of fibroblast proliferation | 7 | 0.005 | 4.59E-02 | FBLN1/PDGFRA/PDGFRB/AQP1/CD74/ANXA2/FN1 |
| BP | GO:0048663~neuron fate commitment | 4 | 0.003 | 4.66E-02 | BMP4/ID2/PAX7/PAX6 |
| BP | GO:0048514~blood vessel morphogenesis | 4 | 0.003 | 4.66E-02 | COL4A1/CYP1B1/ID1/SERPINF2 |
| BP | GO:0021537~telencephalon development | 4 | 0.003 | 4.66E-02 | BMP4/LHX1/SIX3/OXTR |
| BP | GO:0030224~monocyte differentiation | 4 | 0.003 | 4.66E-02 | BMP4/IFI16/MYH9/CSF1R |
| BP | GO:0044849~estrous cycle | 4 | 0.003 | 4.66E-02 | EGR1/ANXA1/OXTR/PTN |
| CC | GO:0031012~extracellular matrix | 48 | 0.037 | 4.10E-13 | ASPN/FMOD/HIST1H4L/LTBP2/LUM/LTBP4/VIM/CLU/COL3A1/DCN/MMP2/PCOLCE/MMRN2/SERPINE2/CD93/COL7A1/COL6A5/TGFBI/COL6A3/COL6A1/LAMB1/LOXL1/THBS2/DPT/SPON1/FN1/COL4A2/COL4A1/LGALS1/EFEMP1/FBN1/COL15A1/MGP/MYH9/ECM1/COL5A2/FLNB/MMP13/ANXA2/VWF/LAMA4/FBLN1/COL14A1/SERPINF1/FBLN2/FBLN5/COL1A2/VCAN |
| CC | GO:0005615~extracellular space | 126 | 0.098 | 4.11E-13 | S100A4/LTBP2/LTBP4/PRTG/GGT1/TNFSF13/MMP2/CXCL12/TNFSF18/MMRN2/APOA4/PTGIS/SERPINE2/CDNF/TGFBI/LOX/CFI/KRT85/SPON1/CLCA1/SPARCL1/CST3/IL25/SERPING1/CST1/FGF21/IL21/PTHLH/SCGB2A1/THBD/SERPINF1/CPE/SERPINF2/TF |

| | | | | | |
|---|---|---|---|---|---|
| | | | | | PI/COL1A2/CPD/CLU/PPT1/IL33/SERPINH1/TIMP1/IFNA4/ANGPTL1/LAMB1/SRGN/SFTPB/PLTP/FN1/BMP4/LGALS1/EFEMP1/S100A11/PPFIBP2/ECM2/ECM1/OMD/TNFSF10/COL14A1/APOL1/SFRP4/CPB1/NRP1/LY86/FAM3B/IGFBP6/LRRC17/RTN3/CFHR3/IL4R/HMOX1/MSN/LOXL1/CTBS/GOLM1/STX4/CLIC1/CBR3/MMP13/SLIT2/STOM/CTSK/GHRH/PLXDC1/CTSO/NPPB/VCAN/RNPEP/FMOD/WNT16/C3/LUM/CPQ/CABP1/COL3A1/DCN/PCOLCE/VCAM1/PRSS8/TNFRSF1A/CD9/COL7A1/COL6A3/KRT2/PTN/DPT/ACTB/KLK6/CES3/PNLIPRP2/FBN1/COL15A1/ANXA1/DPYSL3/SPARC/CCL16/ANXA2/NBL1/AFP/FBLN1/FBLN5/SULF1/CMTM8/LIPG/IFNA14/ALOX5/CD14 |
| CC | GO:0005576~extracellular region | 141 | 0.110 | 1.85E-12 | POTEKP/S100A7/C9ORF47/LTBP4/TNFSF13/JAG1/MMP2/CXCL12/APOA4/OLFML1/BDNF/DSPP/SERPINE2/RNASE7/OSCAR/TGFBI/CFI/LOX/LINC00305/HHIPL2/PMCH/CST3/IL25/HLA-C/SERPING1/FGF21/FGF20/C1QA/PTHLH/WFDC8/C1QB/SERPINF1/SERPINF2/TMPRSS11E/TFPI/COL1A2/MST1L/PLA2G2F/FGFR1/HIST1H4L/FGFR4/CLU/PAMR1/PPT1/IL33/EPHB1/TIMP1/ITGBL1/F13B/C1ORF54/C1ORF56/CRB1/IFNA4/C7ORF69/LAMB1/SRGN/SFTPB/PLTP/FN1/BMP4/EFEMP1/ECM1/APOL3/OMD/TNFSF10/LAMA4/UACA/LAMA3/APOL1/COL14A1/SFRP4/C11ORF44/NXPE1/FAM3B/IGFBP6/CFHR2/CASP4/CLUL1/ITIH5/CASP1/LOXL1/PATE1/LAIR2/INHA/MMP13/SLIT2/CD163/GZMM/CTSK/CHRDL1/SIGLEC6/GHRH/PLXDC1/NPPB/VCAN/TMSB4X/TREM1/ADAM12/RNPEP/SMR3A/C7/FMOD/WNT16/C3/LUM/COL3A1/C1R/C1S/DCN/CPN2/ISLR/PRSS8/TNFRSF1A/COL7A1/CRISPLD2/COL6A5/COL6A3/PYCARD/COL6A1/THBS2/CRIM1/KLK6/COL4A2/COL4A1/PNLIPRP2/SVEP1/RNASE6/FBN1/COL15A1/ANXA1/VWCE/SPARC/CCL16/COL5A2/VWF/FBLN1/FBLN2/FBLN5/LIPG/IFNA14/CD14 |
| CC | GO:0070062~extracellular exosome | 210 | 0.164 | 1.34E-11 | S100A4/POTEKP/LTBP2/S100A7/LTBP4/PDLIM2/GGT1/CD53/AQP1/CD44/OSCAR/RAB25/CFI/DDAH2/DDAH1/FTL/CLCA4/MYH3/SERPING1/COLEC12/H2AFJ/MYH9/PCLO/C1QA/MGAT1/C1QB/SERPINF1/SERPINF2/BHMT/RYR1/PDGFRB/VSIG4/SH3GL2/HLA-DRA/NAGLU/GNAI3/FCER2/PPT1/TAGLN2/ASL/SERPINH1/CD74/EPHB1/KRT24/SEMA5A/STX12/PABPC1/LAMB1/HPCAL1/LGALS1/SLAMF6/S100A11/S100A10/PCDH1 |

| | | | | | |
|---|---|---|---|---|---|
| | | | | | 2/ECM1/LAMA4/COL14A1/LAMA3/DUSP26/LASP1/CD209/MARCKS/PDIA3/FAM3B/RTN3/ACTR2/CFHR3/TKFC/CAP1/DPP7/GOLM1/STX4/PEPD/ICAM2/CLIC1/RFTN1/FLNB/SLIT2/ANXA2P2/SLC26A4/EPB41L2/MTMR11/GNAQ/GNB1/GPA33/RNPEP/C7/RAB3D/C3/CPN2/PCOLCE/CRISPLD2/FAT4/COL6A3/COL6A1/CES3/SHMT2/COL15A1/FZD4/MXRA5/FBLN1/FBLN2/FBLN5/FCGR2A/CD14/CDH11/PGK2/PLXNA1/IQGAP2/TNFSF13/CXCL12/IQGAP1/MMRN2/APOA4/DAB2/GP6/RNASE7/TGFBI/ITCH/BST2/SPARCL1/CST3/HLA-A/MGP/HLA-C/HLA-B/NPEPPS/HLA-E/ARPC1B/CPE/VAMP8/PRCP/COL1A2/CPD/HIST1H4L/IFITM3/CLU/ITGB2/ARPC5/TIMP1/REG1B/GALM/ARPC2/NDRG1/ANGPTL1/ARHGDIB/FN1/CPNE6/EFEMP1/ITGA1/TOMM40/ENDOD1/SAFB2/PSMB9/P2RX4/TNFSF10/OMD/UACA/SLC13A2/CYFIP1/SPTBN1/RAP1B/FABP5/KCTD12/IGFBP6/RPS2/HLA-DMA/PTMA/AKR1C3/MSN/TUBA1B/CTBS/ALK/PCDHGB5/STOM/SCIN/XYLB/FRK/CPQ/LUM/VIM/C1R/C1S/PLPP3/PLBD2/VCAM1/ISLR/PRSS8/CD9/KRT2/MYOF/CRIM1/DPT/ACTB/DCTD/NOX3/RNASE1/COL4A2/LIPA/RNASE6/FBN1/ANXA1/KLK1/TPMT/ANXA4/ANXA2/VWF/CYP4A11/CUL4B/MUC19 |
| CC | GO:0009986~cell surface | 65 | 0.051 | 2.46E-11 | NRP1/PDIA3/TRPV2/ADGRF5/IQGAP2/CD53/LPAR1/CYP2W1/HLA-DMA/TNFSF18/GHRHR/SDC3/APOA4/GP6/CD44/CD93/ROBO1/UNC5D/STX4/BST2/HLA-A/GRIN2A/HLA-C/HLA-B/HLA-E/SLIT2/HLA-F/THBD/SERPINF2/TFPI/PDGFRB/GPA33/HLA-DPA1/ADGRA2/HLA-DRA/AOC3/CLU/ITGB2/CD74/CDH5/VCAM1/TNFRSF1A/FCER1G/PTN/HLA-DPB1/TYROBP/CD28/CSF1R/MRC1/FZD9/LGALS1/ITGA1/ANXA1/TSPAN14/CD1B/SPARC/TPBG/ANXA4/FZD4/DCSTAMP/ANXA2/CD209/SULF1/SFRP4/LIPG |
| CC | GO:0005578~proteinaceous extracellular matrix | 41 | 0.032 | 1.64E-10 | ASPN/FMOD/WNT16/LTBP2/LUM/LTBP4/MMP2/TIMP1/DSPP/CRISPLD2/COL6A5/IMPG1/EMID1/TGFBI/COL6A3/LOX/DPT/SPON1/FN1/BMP4/SPARCL1/LGALS1/EFEMP1/FBN1/COL15A1/MGP/SPARC/ECM2/MMP13/COL5A2/ECM1/SLIT2/VWF/ADAMTS6/OMD/FBLN1/COL14A1/FBLN2/FBLN5/COL1A2/VCAN |
| CC | GO:0005604~basement membrane | 19 | 0.015 | 2.67E-08 | COL4A1/FBN1/CST3/CASK/SPARC/ANXA2/ANXA2P2/MMRN2/TIMP1/FBLN1/LAMA4/LAMA3/COL7A1/SERPINF1/TGFBI/PTN/LAMB1/THBS2/LOXL1 |

| | | | | | |
|---|---|---|---|---|---|
| CC | GO:0005886~plasma membrane | 263 | 0.205 | 1.60E-07 | ADCY8/GGT1/CD53/LPAR1/AQP1/GLDC/CDH22/LILRA1/MALL/CD44/CHRNA9/S1PR5/OSCAR/ADAM7/RECK/OR2S2/CLCA1/CLCA4/EFNB2/MDGA2/GRIN2A/PIM1/COLEC12/MYH9/LPAR6/FER1L5/RYR1/TFPI/PDGFRA/PDGFRB/HLA-DPA1/ADGRA2/DOC2B/BTN3A3/BTN3A2/DBN1/SH3GL2/HLA-DRA/GNAI3/FCER2/ASAP2/RAB40C/RABEPK/FCGRT/GPR143/CD74/EPHB1/SEMA5A/SLC29A2/FCER1G/AHNAK2/HLA-DPB1/TYROBP/KIR2DS5/SLAMF6/PCDH12/GAS1/PTPN12/BTLA/GNGT1/GGT5/KCNJ6/RGS4/ETS2/CD209/RGS7/CHRNB3/SMURF2/MARCKS/OR10A5/NRP1/GLRA1/OR1A2/TRPV2/LY86/GPR82/UNC93A/GPR88/OR7E24/RTN3/SLC24A2/MC5R/STRA6/CAP1/IL13RA1/KCNG1/STX4/PIK3C2G/LTBR/ARHGEF7/ICAM2/CACNG2/CLIC1/RFTN1/FLNB/SLIT2/CD163/EPB41L2/SLC26A4/PJA2/CACFD1/GRM3/GNAQ/LAX1/GNB1/PLXDC1/KCNH8/ADAM12/RNPEP/CLDN16/HLA-DQB1/RAB3D/C3/FFAR1/GNG11/CLDN14/FAT4/CLEC2B/CD28/FZD9/NAT2/CD1B/CLDN20/FZD4/FCGR2B/CDH18/RASSF1/FCGR2A/CD14/ADGRL4/CDH11/QRFPR/PLXNA1/TIRAP/CASK/OR1J4/JAG1/CYP2W1/MMP2/TNFSF18/IQGAP1/GHRHR/SLC7A7/DAB2/ANK1/GP6/ROBO1/TIAM1/GPR45/SLC2A2/C14ORF180/TGFBI/UNC5D/ITCH/BST2/HLA-A/RRP8/HLA-C/HLA-B/NPEPPS/HLA-E/HLA-G/HLA-F/GABRR2/NCAM2/THBD/CPE/VAMP8/PRCP/CPD/CLIP3/KIR2DL3/FGFR1/C3AR1/SLC38A3/FGFR4/SNAP91/DRD3/IFITM2/DRD5/IFITM3/ITGB2/FXYD6/KCNS3/AMER1/OR10C1/ADAM30/NDRG1/TRAF4/CSF1R/MRC1/HCN2/PTPN2/KCNB2/SYT11/CPNE6/GABRA5/CACNA1I/ITGA1/SLCO2B1/MISP/P2RX4/SLC13A2/RAP1B/CACNA1E/PTPN1/DIO1/SLC13A4/ADRA1D/CLDN8/TACR1/ADGRF5/SDC3/MBP/SPRY1/CD93/PAK3/HMOX1/CXCR6/MSN/OR8G1/PTGER2/PTGER4/SIGLEC15/PCDHGB5/TREM1/AOC3/DCC/CD244/PTCHD1/CALD1/VIM/CABP1/OXTR/ESYT1/PLPP3/CDH5/PRSS8/VCAM1/CD9/TNFRSF1A/RGS10/CD4/TAS2R45/INPP5D/MYOF/CRIM1/ACTB/NOX3/IL2RB/NOX1/OR10H1/ANXA1/TSPAN14/CD99/SPARC/ANXA4/ANXA2/CYBB/SULF1/ATP8A2/SCN4B/PPP1R13B |
| CC | GO:0005581~collagen trimer | 19 | 0.015 | 3.16E-07 | COL3A1/COL15A1/COLEC12/SERPINH1/MMP13/COL5A2/PCOLCE/TIMP1/C1QA/C1 |

| | | | | | |
|---|---|---|---|---|---|
| | | | | | QB/COL14A1/COL7A1/COL6A5/EMID1/COL6A3/COL1A2/COL6A1/SCARA3/LOX |
| CC | GO:0071556~integral component of lumenal side of endoplasmic reticulum membrane | 11 | 0.009 | 5.38E-07 | HLA-DQB1/HLA-A/HLA-C/HLA-DPA1/HLA-B/HLA-DPB1/HLA-E/CD74/HLA-G/HLA-DRA/HLA-F |
| CC | GO:0005887~integral component of plasma membrane | 109 | 0.085 | 8.37E-07 | QRFPR/CALHM2/PLXNA1/CD53/LPAR1/JAG1/AQP1/SLC7A7/GP6/CD44/CHRNA9/ROBO1/SLC2A2/TYRO3/CLCA1/CLCA4/BST2/SLC22A24/EFNB2/HLA-A/GRIN2A/HLA-C/HLA-B/GABRR2/THBD/LPAR6/TMPRSS11E/PDGFRA/RYR1/HLA-DPA1/GPR52/KIR2DL3/HLA-DRA/FGFR1/C3AR1/FGFR4/SLC38A3/DRD3/DRD5/FCER2/FXYD5/EPHB1/LAPTM5/SLC29A2/FCER1G/SLC30A3/TYROBP/CSF1R/MRC1/HCN2/GUCY2F/KIR2DS5/SYT11/GABRA5/PCDH12/KIR2DS3/SLCO2B1/TPBG/DCSTAMP/BFAR/P2RX4/TNFSF10/NTRK2/SLC13A2/SLC13A4/ADRA1D/SLC5A4/GLRA1/TACR1/TRPV2/GJA4/GPR88/IL4R/SLC24A2/CXCR6/MC5R/GOLM1/PTGER2/LTBR/PTGER4/GPR19/ICAM2/ALK/CD163/SLC26A4/STOM/GRM3/SIGLEC6/CSPG4P5/GPA33/KCNH8/TM4SF1/TFR2/FFAR1/OXTR/PLPP3/TNFRSF1A/CD9/CLEC2B/CD28/PTPRB/IL2RB/TMBIM6/TSPAN14/OR10H1/CD99/PRPH2/FZD4/CYBB |
| CC | GO:0042612~MHC class I protein complex | 7 | 0.005 | 4.52E-06 | HLA-DQB1/HLA-A/HLA-C/HLA-B/HLA-E/HLA-G/HLA-F |
| CC | GO:0042613~MHC class II protein complex | 9 | 0.007 | 4.79E-06 | HLA-DQB1/HLA-A/HLA-C/HLA-DPA1/HLA-DPB1/HLA-DMB/HLA-DMA/CD74/HLA-DRA |
| CC | GO:0045121~membrane raft | 25 | 0.020 | 5.50E-05 | GNAI3/BST2/ITGA1/S100A10/PPT1/RFTN1/SERPINH1/IQGAP1/EPHB1/ANXA2/EFHD2/STOM/TNFRSF1A/MALL/STX12/LAX1/SULF1/CMTM8/SMURF2/CD4/RAP1B/CLIP3/INPP5D/CD14/FAIM2 |
| CC | GO:0012507~ER to Golgi transport vesicle membrane | 11 | 0.009 | 1.56E-04 | HLA-DQB1/HLA-A/HLA-C/HLA-DPA1/HLA-B/HLA-DPB1/HLA-E/CD74/HLA-G/HLA-DRA/HLA-F |
| CC | GO:0016021~integral | 294 | 0.230 | 3.63E-04 | CYP3A5/RAD51B/SSMEM1/B3GALT5/ADCY8/LOC441081/GGT1/CD53/LPAR1/AQP1/ |

| | | | | | |
|---|---|---|---|---|---|
| | component of membrane | | | | ACBD4/CDH22/DSPP/BDNF/LILRA1/MALL/CD44/MAP3K9/S1PR5/OSCAR/MYRFL/CREB3L2/SCD5/ADAM7/HIGD1B/TYRO3/OR2S2/MDGA2/GRIN2A/UBE2J1/ABCC13/COLEC12/PCLO/MGAT1/LHFPL2/LPAR6/TMPRSS11E/FER1L5/RYR1/PDGFRB/LNPK/HLA-DPA1/AMFR/ADGRA2/VSIG4/BTN3A3/BTN3A2/HLA-DRA/CYP1B1/SMIM11A/FCER2/FCGRT/GPR143/CD74/EPHB1/SEMA5A/STX12/TEDDM1/FCER1G/HLA-DPB1/TRAM1/TYROBP/CYP46A1/KIR2DS5/SLAMF6/S100A10/PCDH12/GAS1/KIR2DS3/DCSTAMP/SYNGR4/BTLA/GGT5/BFAR/IFI27/C1ORF115/APOL1/CD209/SFRP4/NTRK2/CHRNB3/SMURF2/NXPE1/OR10A5/NRP1/GLRA1/OR1A2/TRPV2/GPR82/UNC93A/TMEM161A/LTC4S/GJA4/RTN1/OR7E24/RTN3/ATAD3A/TMEM108/POMGNT2/DNAJC16/CHST12/IL4R/VKORC1/MC5R/STRA6/CHST15/IL13RA1/PIEZO2/STX4/ICAM2/ERLIN1/MMP13/FLNB/LRRC25/CD163/SLC26A4/CACFD1/GRM3/LAX1/PLXDC1/ORMDL2/ADAM12/SERP1/CLDN16/HLA-DQB1/FUT9/MTDH/HSD17B2/TFR2/FFAR1/CLDN14/CFAP54/GALNT10/FAT4/LRRTM2/FBXW12/GALNT11/FZD9/MOGAT2/NAT2/COL15A1/CD1B/CLDN20/FZD4/FCGR2B/CDH18/CFAP47/BNIP3L/CMTM8/FCGR2A/DRAM1/SSR2/ADGRL4/CDH11/QRFPR/LRTM1/PRTG/LRTM2/TNFSF13/OR1J4/JAG1/TNFSF18/DSE/GHRHR/SLC7A7/PTGIS/GPR45/SLC2A2/C14ORF180/COQ8A/UNC5D/RPN2/LEPROT/TMPRSS15/LRRN4CL/BST2/SLC22A24/HLA-A/HLA-C/AJAP1/HLA-B/HLA-E/HLA-G/HLA-F/GABRR2/PNPLA7/NCAM2/VAMP8/GPR52/CPD/KIR2DL3/ARL6IP1/GAL3ST2/NETO2/FGFR1/C3AR1/TMEM212/SLC38A3/FGFR4/LST1/IFITM2/IFITM3/IL33/FXYD5/FXYD6/ABCA6/KCNS3/TCP11/CRB1/ABHD16A/RNFT2/OR10C1/ADAM30/SLC30A3/CSF1R/RNF13/PTPN2/GABRA5/TOMM40/SLCO2B1/ADGRG4/TNFSF10/UCP3/FAAH/SLC13A2/SLC35G3/SCARA3/PTPN1/DIO1/SLC13A4/ADRA1D/NYNRIN/CLDN8/IER3/SLC5A4/NDST1/TACR1/ADGRF5/HLA-DMB/HLA-DMA/SDC3/CD93/HMOX1/CXCR6/PIGC/OR8G1/BTNL8/SVOP/PTGER2/GPR19/PTGER4/CHST4/HEPACAM2/ALK/TMEM75/SIGLEC15/PCDHGB5/SIGLEC6/TREM1/TM4SF1/FAIM2/AOC3/DCC/CD244/PTCHD1/HS3ST2/OXTR/ESYT1/CDH5/SEC63/PRSS8/VCAM1/CD9/TNFRSF1A/CD4/TAS2R45/MYOF/CRIM1/ |

| | | | | | |
|---|---|---|---|---|---|
| | | | | | HHATL/MS4A4A/HS3ST3A1/TMBIM6/NOX1/TSPAN14/OR10H1/CD99/PRPH2/GIMAP1/CYP4A11/CYBB/C3ORF52/ATP8A2/SCN4B/CHRNA10 |
| CC | GO:0005925~focal adhesion | 35 | 0.027 | 6.29E-04 | NRP1/PDIA3/SRCIN1/S100A7/VIM/PDLIM2/CASK/ARPC5/RPS2/IQGAP1/ACTR2/CD9/DAB2/CD44/ARPC2/CAP1/MSN/PABPC1/ACTB/ARHGEF7/EFNB2/ANXA1/ITGA1/CD99/CSRP2/MISP/MYH9/FLNB/PTPN12/EPB41L2/ARPC1B/LASP1/CYFIP1/PDGFRB/MARCKS |
| CC | GO:0031093~platelet alpha granule lumen | 10 | 0.008 | 1.13E-03 | ISLR/VWF/SERPINF2/CLU/SERPING1/TMSB4X/SPARC/SRGN/FN1/TIMP1 |
| CC | GO:0005788~endoplasmic reticulum lumen | 20 | 0.016 | 2.29E-03 | CES3/COL4A2/COL4A1/PDIA3/COL3A1/COL15A1/CYP2W1/COL5A2/SERPINH1/APOA4/COL14A1/COLGALT2/COL7A1/COL6A3/COL1A2/COL6A1/CD4/FKBP10/RCN1/SPON1 |
| CC | GO:0005783~endoplasmic reticulum | 59 | 0.046 | 2.48E-03 | CLDN8/GLRA1/PDIA3/B3GALT5/S100A7/LTC4S/DSE/RTN3/CASP4/PTGIS/POMGNT2/CDNF/ELOVL4/HMOX1/EMID1/CREB3L2/RPN2/ZFYVE1/HLA-A/GRIN2A/CST3/HLA-C/ERLIN1/HLA-B/HLA-F/STOM/PNPLA7/ARCN1/TFPI/ORMDL2/SERP1/AOC3/FGFR4/MTDH/CPQ/CLU/SERPINH1/CLDN14/SEC63/VCAM1/A1CF/PYCARD/PTN/TRAM1/HHATL/KLK6/MOGAT2/CYP46A1/PTPN2/TMBIM6/TPBG/VWF/RAB32/BFAR/SULF1/BNIP3L/PTPN1/RCN1/SSR2 |
| CC | GO:0030669~clathrin-coated endocytic vesicle membrane | 8 | 0.006 | 3.09E-03 | HLA-DQB1/CD9/HLA-DPA1/HLA-DPB1/FZD4/SH3GL2/CD74/HLA-DRA |
| CC | GO:0005765~lysosomal membrane | 25 | 0.020 | 3.43E-03 | HLA-DQB1/GNAI3/IFITM3/HLA-DMB/GPR143/HLA-DMA/CD74/BORCS8/DAB2/LAPTM5/COL6A1/HLA-DPB1/SLC30A3/RNF13/SPHK2/CD1B/ANXA2/PNPLA7/P2RX4/GNAQ/GNB1/VAMP8/HLA-DPA1/DRAM1/HLA-DRA |
| CC | GO:0072562~blood microparticle | 16 | 0.012 | 6.48E-03 | ACTB/C3/CLU/SERPING1/C1R/C1S/CLIC1/CPN2/APOA4/STOM/C1QB/CFHR3/APOL1/SERPINF2/MSN/FN1 |
| CC | GO:0005589~collagen type VI trimer | 3 | 0.002 | 6.69E-03 | COL6A3/COL6A1/DCN |

| | | | | | |
|---|---|---|---|---|---|
| CC | GO:0030670~phagocytic vesicle membrane | 9 | 0.007 | 6.92E-03 | RAB32/CYBB/ATG12/HLA-A/HLA-C/HLA-B/HLA-E/HLA-G/HLA-F |
| CC | GO:0042383~sarcolemma | 11 | 0.009 | 7.44E-03 | VCAM1/ANK1/COL6A3/ANXA1/RYR1/AHNAK2/COL6A1/PPP3CA/AQP1/GHRHR/ANXA2 |
| CC | GO:0043202~lysosomal lumen | 11 | 0.009 | 7.44E-03 | FMOD/OMD/NAGLU/LUM/PDGFRB/VCAN/PPT1/DCN/CD74/SDC3/PLBD2 |
| CC | GO:1903561~extracellular vesicle | 8 | 0.006 | 9.46E-03 | MGAT1/CD9/OLFML3/SERPINE2/FBLN2/GNB1/COL6A3/ITGB2 |
| CC | GO:0042588~zymogen granule | 3 | 0.002 | 1.30E-02 | GNAI3/RAB3D/SRGN |
| CC | GO:0030666~endocytic vesicle membrane | 9 | 0.007 | 1.34E-02 | HLA-DQB1/CD9/COLEC12/HLA-DPA1/CACNG2/HLA-DPB1/CD74/HLA-DRA/CD163 |
| CC | GO:0005885~Arp2/3 protein complex | 4 | 0.003 | 1.75E-02 | ACTR2/ARPC1B/ARPC2/ARPC5 |
| CC | GO:0043020~NADPH oxidase complex | 4 | 0.003 | 1.75E-02 | NOX3/CYBB/NCF2/NOX1 |
| CC | GO:0005794~Golgi apparatus | 56 | 0.044 | 1.95E-02 | SNCB/ZC3HAV1/B3GALT5/IGFBP6/DSE/SPRY1/CD44/EMID1/LEPROT/FBXL14/DPP7/GOLM1/BST2/LIG1/HLA-A/HLA-C/HLA-B/HEPACAM2/MECOM/RUBCN/PTHLH/CPE/LAX1/ARCN1/BTN3A3/AOC3/GAL3ST2/FGFR4/FUT9/LST1/GNAI3/USP8/CPQ/CLU/PPT1/GPR143/PLPP3/KCNS3/PLAGL1/VCAM1/GOLGA6L2/ARPC2/PLIN3/OBSL1/KRT2/SRGN/FZD9/PLA2G4A/CYBB/KCNJ6/DUSP26/ID1/SULF1/LIPG/ATP8A2/CD14 |
| CC | GO:0072557~IPAF inflammasome complex | 3 | 0.002 | 2.09E-02 | NLRC4/CASP4/CASP1 |
| CC | GO:0031091~platelet alpha granule | 4 | 0.003 | 2.70E-02 | VWF/SERPINE2/SPARC/THBS2 |
| CC | GO:0000139~Golgi membrane | 40 | 0.031 | 2.83E-02 | HLA-DQB1/FUT9/LST1/NDST1/B3GALT5/HS3ST2/CABP1/DSE/CD74/RTN3/TNFRSF1A/GALNT10/MALL/STX12/COL7A1/CHST12/CHST15/HLA-DPB1/LEPROT/GALNT11/SRGN/RNF13/HS3ST3A1/HLA-A/HLA-C/CHST4/HLA-B/HEPACAM2/HLA-E/HLA-G/GIMA |

| | | | | | |
|---|---|---|---|---|---|
| | | | | | P1/HLA-F/MGAT1/PJA2/ARCN1/ATP8A2/HLA-DPA1/SCARA3/SH3GL2/HLA-DRA |
| CC | GO:0097169~AIM2 inflammasome complex | 3 | 0.002 | 3.04E-02 | CASP4/PYCARD/CASP1 |
| CC | GO:0030658~transport vesicle membrane | 6 | 0.005 | 3.41E-02 | HLA-DQB1/CPE/HLA-DPA1/HLA-DPB1/CD74/HLA-DRA |
| MF | GO:0005518~collagen binding | 18 | 0.014 | 1.37E-09 | ASPN/SPARCL1/LUM/ITGA1/SPARC/DCN/MMP13/SERPINH1/PCOLCE/VWF/CTSK/DSPP/GP6/COL14A1/CD44/TGFBI/SRGN/FN1 |
| MF | GO:0005201~extracellular matrix structural constituent | 18 | 0.014 | 8.71E-09 | COL4A2/COL4A1/LUM/COL3A1/FBN1/COL15A1/MGP/COL5A2/FBLN1/LAMA4/DSPP/COL14A1/FBLN2/IMPG1/COL1A2/CD4/VCAN/LAMB1 |
| MF | GO:0005509~calcium ion binding | 69 | 0.054 | 2.85E-08 | ASPN/S100A4/SNCB/LTBP2/S100A7/AIF1/LTBP4/JAG1/IQGAP1/EFHD2/CDH22/DSPP/CD93/SLC24A2/PLS3/SPARCL1/MGP/PCLO/MMP13/SLIT2/PCDHGB5/ANXA2P2/THBD/SCIN/RYR1/VCAN/FKBP10/DOC2B/PLA2G2F/AOC3/MYL7/CABP1/PAMR1/C1R/C1S/NECAB3/CDH5/CRB1/FAT4/EFCAB1/PPP3CA/THBS2/HPCAL1/PNLIPRP2/SVEP1/CPNE6/SYT11/CAPN9/FBN1/EFEMP1/S100A11/ANXA1/VWCE/S100A10/PCDH12/SPARC/ANXA4/ANXA2/FBLN1/PLA2G4A/EFHB/FBLN2/FBLN5/CDH18/SULF1/CACNA1E/RCN1/ADGRL4/CDH11 |
| MF | GO:0042605~peptide antigen binding | 11 | 0.009 | 3.11E-07 | HLA-DQB1/CD209/HLA-A/HLA-C/HLA-DPA1/FCGRT/HLA-B/HLA-DPB1/HLA-E/HLA-DRA/HLA-F |
| MF | GO:0048407~platelet-derived growth factor binding | 6 | 0.005 | 8.38E-05 | COL4A1/COL3A1/PDGFRA/COL1A2/PDGFRB/COL6A1 |
| MF | GO:0005178~integrin binding | 15 | 0.012 | 4.08E-04 | VCAM1/CD9/VWF/FBLN1/PTPN2/FCER2/FBLN5/LTBP4/ICAM2/COL3A1/TGFBI/FBN1/ECM2/PLPP3/FN1 |
| MF | GO:0032395~MHC class II receptor activity | 6 | 0.005 | 4.65E-04 | HLA-DQB1/HLA-C/HLA-DPA1/HLA-DPB1/HLA-DMA/HLA-DRA |
| MF | GO:0038085~vascular endothelial growth factor | 4 | 0.003 | 9.72E-04 | NRP1/PDGFRA/PDGFRB/PTN |

| | | | | | |
|---|---|---|---|---|---|
| | binding | | | | |
| MF | GO:0004859~phospholipase inhibitor activity | 5 | 0.004 | 1.24E-03 | SNCB/ANXA1/ANXA4/ANXA2/ANXA2P2 |
| MF | GO:0005102~receptor binding | 29 | 0.023 | 5.11E-03 | FRK/C3/IGFBP6/TNFSF13/TNFSF18/CXCL12/CDH5/SERPINE2/ANGPTL1/MSN/LEPROT/TYROBP/ANXA1/HLA-A/SIX3/ITGA1/HLA-B/INHA/FGF20/HLA-E/HLA-G/HLA-F/P2RX4/TNFSF10/LAMA4/LAMA3/NPPB/PDGFRB/CHRNA10 |
| MF | GO:0005200~structural constituent of cytoskeleton | 13 | 0.010 | 5.66E-03 | ACTB/VIM/ARPC5/EPB41L2/CYLC1/ARPC1B/ACTR2/ANK1/ARPC2/SPTBN1/KRT2/MSN/TUBA1B |
| MF | GO:0004867~serine-type endopeptidase inhibitor activity | 12 | 0.009 | 5.92E-03 | RECK/WFDC8/SERPINE2/COL7A1/SERPINF1/SERPINF2/COL6A3/TFPI/ITIH5/SERPING1/SERPINH1/CRIM1 |
| MF | GO:0046977~TAP binding | 3 | 0.002 | 6.45E-03 | HLA-A/HLA-C/HLA-B |
| MF | GO:0002020~protease binding | 12 | 0.009 | 7.99E-03 | VWF/SERPINF2/CST3/PYCARD/RYR1/CST1/ECM1/ANXA2/FN1/MBP/TIMP1/CD28 |
| MF | GO:0001077~transcriptional activator activity, RNA polymerase II core promoter proximal region sequence-specific binding | 21 | 0.016 | 8.25E-03 | EGR1/EPAS1/MAFB/CEBPG/PAX6/LEF1/ELK3/POU1F1/TCF21/GATA2/FLI1/MEOX2/EBF2/POU2F3/IRF1/CREB3L2/NEUROD1/TCF4/RUNX2/KLF4/NFIB |
| MF | GO:0008201~heparin binding | 16 | 0.012 | 8.72E-03 | BMP4/FMOD/FGFR1/FGFR4/NRP1/LTBP2/FBN1/ECM2/PCOLCE/SLIT2/SERPINE2/CRISPLD2/LIPG/PTN/THBS2/FN1 |
| MF | GO:0035240~dopamine binding | 4 | 0.003 | 9.77E-03 | DRD3/DRD5/GPR52/GPR143 |
| MF | GO:0048365~Rac GTPase binding | 7 | 0.005 | 1.05E-02 | NCF2/TIAM1/NOX1/CYFIP1/IQGAP2/IQGAP1/ARHGDIB |
| MF | GO:0016175~superoxide-generating NADPH oxidase activity | 4 | 0.003 | 1.30E-02 | NOX3/CYBB/NCF2/NOX1 |
| MF | GO:0030881~beta-2-microglobulin binding | 4 | 0.003 | 1.30E-02 | HLA-A/CD1B/FCGRT/HLA-E |

| | | | | | |
|---|---|---|---|---|---|
| MF | GO:0019864~IgG binding | 4 | 0.003 | 1.30E-02 | FCGR2B/FCER1G/FCGRT/FCGR2A |
| MF | GO:0005254~chloride channel activity | 8 | 0.006 | 1.30E-02 | GABRR2/SLC26A4/CLCA1/APOL1/CLCA4/GABRA5/CLIC2/CLIC1 |
| MF | GO:0005262~calcium channel activity | 9 | 0.007 | 1.69E-02 | CACFD1/CHRNA9/SLC24A2/TRPV2/GRIN2A/RYR1/CACNA1E/CACNG2/CHRNA10 |
| MF | GO:0005544~calcium-dependent phospholipid binding | 8 | 0.006 | 1.88E-02 | PLA2G4A/SYT11/ANXA1/DOC2B/PCLO/ANXA4/ANXA2/ANXA2P2 |
| MF | GO:0043425~bHLH transcription factor binding | 5 | 0.004 | 2.12E-02 | TCF21/LMO2/TCF4/RUNX2/TWIST1 |
| MF | GO:0051015~actin filament binding | 13 | 0.010 | 2.21E-02 | AIF1/MYH3/IQGAP2/ARPC5/MYH9/ACTR2/ARPC1B/LASP1/ARPC2/SCIN/CYFIP1/MARCKS/PLS3 |
| MF | GO:0005198~structural molecule activity | 20 | 0.016 | 2.47E-02 | CLDN8/CLDN16/VIM/ANXA1/COL15A1/JAG1/CLDN20/CLDN14/KRT23/KRT24/EPB41L2/CYLC1/NPHP4/ANK1/LAMA3/KRT40/SCARA3/KRT85/LAMB1/TUBA1B |
| MF | GO:0004185~serine-type carboxypeptidase activity | 4 | 0.003 | 2.58E-02 | CPE/PRCP/CPD/DPP7 |
| MF | GO:0008289~lipid binding | 14 | 0.011 | 2.64E-02 | FFAR1/S100A10/LTC4S/ESYT1/ACBD4/APOA4/NME4/CADPS/APOL3/APOL1/TIAM1/SH3GL2/FABP5/PLTP |
| MF | GO:0001588~dopamine neurotransmitter receptor activity, coupled via Gs | 3 | 0.002 | 2.93E-02 | DRD3/DRD5/GPR52 |
| MF | GO:0001102~RNA polymerase II activating transcription factor binding | 6 | 0.005 | 3.19E-02 | IFI27/DUSP26/LMO2/HEY2/NEUROD1/POU1F1 |
| MF | GO:0050840~extracellular matrix binding | 5 | 0.004 | 3.21E-02 | FBLN2/SPARCL1/TGFBI/DCN/SPARC |
| MF | GO:0005515~protein binding | 441 | 0.345 | 3.31E-02 | XRCC2/LTBP2/LTBP4/FLI1/MALL/SERPINE2/MAP3K9/DDAH2/TYRO3/NCF2/GRIN2A/ |

| | | | | | |
|---|---|---|---|---|---|
| | | | | | ZNF501/ZHX3/SERPING1/GEM/MECOM/MYH9/NME4/SERPINF1/TAGLN/SERPINF2/MAPK4/RYR1/TGIF1/SH3GL2/ACAA1/GNAI3/HSD17B14/PPT1/ASL/RCC1/NAA35/NPHP4/IFNA4/AHNAK2/OBSL1/PABPC1/TCF4/SPOP/SPHK2/MAGEB2/GAS1/MAGEB4/PRPSAP1/FAM131B/DCSTAMP/BFAR/GNGT1/KCNJ6/APOL1/ZNF217/DUSP26/LASP1/CD209/TSGA10/SMURF2/PDIA3/GLRA1/LTC4S/RTN1/GTSE1/RTN3/POMGNT2/TKFC/IL4R/MC5R/FBXL14/IL13RA1/ATF7IP/STX4/PEPD/ARHGEF7/ERLIN1/CLIC2/CLIC1/CACNG2/FLNB/ZNF335/CARD10/LRRC25/CACFD1/IGFN1/GNAQ/GNB1/PLXDC1/KIF25-AS1/ESRP1/VCAN/TMSB4X/TRIM17/C3/TFR2/OFCC1/TRIM10/NECAB3/ZNF34/PCOLCE/COLGALT2/COL7A1/COL6A5/PPP3CA/SHMT2/LMX1B/WWTR1/TRIM22/ZNF165/RASSF1/BNIP3L/SRCIN1/LMO2/LMO4/ZXDC/JAG1/OTUB2/IQGAP1/APOA4/NLRC4/DAB2/CDKN2A/ANK1/ELOVL4/TGFBI/PITPNC1/RPN2/ANKZF1/SPON1/HHIPL2/BST2/SIX3/HLA-A/MGP/TMSB10/RUBCN/THBD/VAMP8/COL1A2/PRCP/HIST1H4L/DRD3/IFITM3/IL33/ARPC5/TIMP1/AMER1/CRB1/ARPC2/TEAD4/TRAF5/TRAF4/ARHGDIB/MRC1/EPAS1/SYT11/CACNA1I/EFEMP1/SUV39H1/SAFB2/HSPBP1/P2RX4/TNFSF10/FAAH/ADRA1D/FABP5/CLDN8/IER3/IER5/NDST1/PAX6/DPH2/HNRNPLL/RPS2/PTMA/MBP/SPRY1/HMOX1/HEY2/CASP1/PTGER4/KIF15/IFI16/SH2D3C/SIGLEC6/MAPRE3/CPSF2/LCP2/DCC/HYPM/VIM/CABP1/DCN/ESYT1/PLPP3/CDH5/SEC63/PRSS8/CD9/A1CF/CD4/INPP5D/GTF3C1/MAF/KLK6/COL4A2/RNASE1/COL4A1/CEBPG/NOX1/FBN1/ANXA1/FAM9B/DPYSL3/ANXA4/ANXA2/CADPS/CYBB/ID2/ID1/UCKL1/ID3/CUL4B/THEMIS2/RCN1/S100A4/RAD51B/PNMA2/S100A7/PDLIM2/GGT1/CD53/LPAR1/AQP1/HEMGN/CD44/RAB25/CFI/TWIST1/FTL/RECK/EMX1/RPUSD3/EFNB2/PIM1/C1QA/C1QB/PRDM4/PDGFRA/PDGFRB/AMFR/PRDM1/ADGRA2/DBN1/FCER2/ASAP2/GPR143/TAGLN2/CD74/EPHB1/STX12/FCER1G/TRAM1/TYROBP/BMP4/KLF6/HPCAL1/LGALS1/FAM111A/S100A11/RYBP/SLAMF6/S100A10/CSRP2/RAD54L/ECM1/PTPN12/LAMA4/ETS2/SFRP4/KLF4/NRP1/GJA4/ARL2BP/ALOX12B/GOLM1/ZFYVE1/PATE1/LTBR/THEG/SLIT2/CD163/GZMM/DACT1/LAX1/NPPB/GPA33/SERP1/CLDN16/USP8/MTDH |

| | | | | | |
|---|---|---|---|---|---|
| | | | | | /ATG12/COL3A1/ZFP36L1/FAT4/PLIN3/THBS2/CD28/SPSB3/IPO11/NAT2/CD1B/SNAI1/FZD4/AFP/RAB32/MEOX2/FCGR2B/ANKRD49/FBLN5/MEX3C/NEUROD1/FCGR2A/DRAM1/CD14/PBX4/TEX11/APOBEC1/ZC3HAV1/CLPB/TIRAP/CASK/MMP2/GHRHR/MMRN2/TOP1/GATA2/GP6/PTGIS/TIAM1/ROBO1/COQ8A/ITCH/LOX/TMPRSS15/PID1/SNRPN/CST3/CST1/RRP8/FGF21/TIMM8A/MAP4K4/MAP4K5/CLIP3/KIR2DL3/ARL6IP1/GAL3ST2/FGFR1/MYL7/FGFR4/SNAP91/CLU/ITGB2/ELK3/C2ORF50/FXYD6/LAPTM5/ABHD16A/ANKIB1/NDRG1/SLC30A3/RUNX2/SRGN/FN1/RNF13/PTPN2/MAFB/CPNE6/ITGA1/TOMM40/HEATR1/PSMB9/PPIE/UACA/UCP3/SPTBN1/CYFIP1/DNMT1/RAP1B/PTPN1/SCARA3/HPS5/TACR1/TTPA/TDO2/CD93/PAK3/KRT40/MSN/TUBA1B/EGR1/BTNL8/KHDRBS3/LAIR2/LEF1/HEPACAM2/INHA/ALK/TIMM44/STOM/CTSK/ZMIZ2/AOC3/UTP3/FRK/CD244/CALD1/LUM/C1R/C1S/C3ORF62/BORCS8/TNFRSF1A/POU2F3/DYDC1/PYCARD/KRT2/GEMIN6/MYOF/PTPRB/DCTD/ACTB/IL2RB/TMBIM6/SUN2/SPARC/SH3BP5/VWF/NBL1/C3ORF52/IRF1/PPP1R13B/ALOX5 |
| MF | GO:0004181~metallocarboxypeptidase activity | 5 | 0.004 | 3.63E-02 | PEPD/AGBL1/CPE/CPD/CPB1 |
| MF | GO:0023026~MHC class II protein complex binding | 4 | 0.003 | 3.70E-02 | HLA-DMB/HLA-DMA/CD74/HLA-DRA |
| MF | GO:0004180~carboxypeptidase activity | 4 | 0.003 | 4.33E-02 | CPE/CPQ/PRCP/CPB1 |
