# Supplementary material for: Identification of the Potential Molecular Mechanism of TGFBI Gene in Persistent Atrial Fibrillation
Source: Comput Math Methods Med. 2022 Nov 8;2022:1643674. doi: 10.1155/2022/1643674 (PMC9666036; doi:10.1155/2022/1643674)
Supplement: Supplementary Materials — Table S1: GO annotation of TGFBI and coexpression genes enriched in AF. Table S2: GO annotation and KEGG pathway TGFBI and coexpression genes enriched in cluster 1. Figure S1: the clusters constructed from the PPI-network. [file 1643674.f1.zip › Table S2. GO annotation and KEGG pathway TGFBI and co-expression genes enriched in Cluster 1.pdf]

Table S2. GO annotation and KEGG pathway TGFBI and co-expression genes enriched in Cluster 1

Category	Term	Count	%	PValue	Genes
BP	GO:0030198~extracellular matrix organization	25	0.126	3.12E-21	COL4A2/COL4A1/LUM/COL3A1/ITGA1/ITGB2/SPARC/DCN/COL5A2/VCAM1/FBLN1/LAMA4/COL14A1/CD44/COL7A1/FBLN5/COL6A3/TGFBI/COL1A2/COL6A1/VCAN/LOX/LAMB1/LOXL1/FN1
BP	GO:0060333~interferon-gamma-mediated signaling pathway	13	0.065	7.59E-13	HLA-DQB1/VCAM1/CD44/HLA-A/IRF1/HLA-C/HLA-DPA1/HLA-B/HLA-DPB1/HLA-E/TRIM22/HLA-G/HLA-F
BP	GO:0030574~collagen catabolic process	12	0.060	5.85E-12	COL4A2/COL4A1/COL7A1/COL6A5/COL3A1/COL6A3/COL1A2/COL15A1/COL6A1/MMP13/MMP2/COL5A2
BP	GO:0060337~type I interferon signaling pathway	12	0.060	5.85E-12	EGR1/IFI27/BST2/IFITM2/IFITM3/HLA-A/IRF1/HLA-C/HLA-B/HLA-E/HLA-G/HLA-F
BP	GO:0030199~collagen fibril organization	10	0.050	3.12E-11	FMOD/COL14A1/SERPINF2/LUM/COL3A1/COL1A2/LOX/SERPINH1/COL5A2/ANXA2
BP	GO:0045087~innate immune response	21	0.106	6.83E-10	FRK/NCF2/BST2/S100A7/LY86/CLU/ANXA1/HLA-C/SERPING1/HLA-B/TRIM10/HLA-E/C1QA/C1QB/CYBB/APOL1/PYCARD/FCER1G/CD14/TYROBP/CSF1R
BP	GO:0006955~immune response	20	0.101	3.11E-09	HLA-DQB1/PTGER4/C3/IFITM2/IFITM3/HLA-A/HLA-C/HLA-B/HLA-DMB/CCL16/HLA-E/HLA-DMA/TRIM22/CXCL12/HLA-F/FCGR2B/CD4/HLA-DPA1/HLA-DPB1/LCP2
BP	GO:0050776~regulation of immune response	14	0.070	3.97E-09	C3/COL3A1/HLA-A/ITGB2/HLA-C/HLA-B/HLA-E/HLA-G/HLA-F/VCAM1/FCGR2B/IRF1/COL1A2/TYROBP
BP	GO:0002480~antigen processing and presentation of exogenous peptide antigen via MHC class I, TAP-independent	6	0.030	5.12E-09	HLA-A/HLA-C/HLA-B/HLA-E/HLA-G/HLA-F
BP	GO:0019882~antigen processing and presentation	9	0.045	1.97E-08	HLA-DQB1/HLA-A/HLA-C/HLA-DPA1/HLA-B/HLA-DPB1/HLA-DMB/HLA-E/HLA-G

BP	GO:0002479~antigen processing and presentation of exogenous peptide antigen via MHC class I, TAP-dependent	9	0.045	5.93E-08	CYBB/NCF2/HLA-A/HLA-C/HLA-B/HLA-E/HLA-G/PSMB9/HLA-F
BP	GO:0007155~cell adhesion	19	0.096	7.17E-08	COL15A1/ITGB2/CXCL12/VCAM1/ISLR/LAMA4/CD44/COL7A1/CTGF/COL6A5/COL6A3/TGFBI/COL6A1/CD4/VCAN/LAMB1/THBS2/FN1/CDH11
BP	GO:0035987~endodermal cell differentiation	7	0.035	8.77E-08	COL4A2/COL7A1/COL6A1/ITGB2/LAMB1/MMP2/FN1
BP	GO:0002474~antigen processing and presentation of peptide antigen via MHC class I	7	0.035	1.72E-07	HLA-DQB1/HLA-A/HLA-C/HLA-B/HLA-E/HLA-G/HLA-F
BP	GO:0007189~adenylate cyclase-activating G-protein coupled receptor signaling pathway	8	0.040	2.06E-07	PTHLH/PTGER2/PTGER4/GNAQ/GHRH/ADCY8/DRD5/GHRHR
BP	GO:0006954~inflammatory response	15	0.075	4.46E-06	C3AR1/PTGER2/C3/AIF1/TACR1/LY86/ANXA1/ITGB2/CCL16/CXCL12/CYBB/CXCR6/PYCARD/CD14/CSF1R
BP	GO:0002504~antigen processing and presentation of peptide or polysaccharide antigen via MHC class II	5	0.025	1.10E-05	HLA-DQB1/HLA-DPA1/HLA-DPB1/HLA-DMB/HLA-DMA
BP	GO:0042102~positive regulation of T cell proliferation	7	0.035	1.18E-05	VCAM1/AIF1/ANXA1/CD4/HLA-DPA1/HLA-DPB1/HLA-DMB
BP	GO:0007229~integrin-mediated signaling pathway	8	0.040	2.17E-05	DAB2/FBLN1/CTGF/COL3A1/ITGA1/FCER1G/ITGB2/TYROBP
BP	GO:0002576~platelet degranulation	8	0.040	2.81E-05	ISLR/SERPINF2/CLU/SERPING1/TMSB4X/SPARC/SRGN/FN1

CC	GO:0031012~extracellular matrix	31	0.156	8.86E-25	FMOD/LUM/COL3A1/CLU/DCN/MMP2/PCOLCE/CD93/COL7A1/COL6A5/COL6A3/TGFBI/COL6A1/LAMB1/LOXL1/THBS2/FN1/COL4A2/COL4A1/LGALS1/COL15A1/COL5A2/MMP13/ANXA2/LAMA4/FBLN1/COL14A1/FBLN2/FBLN5/COL1A2/VCAN
CC	GO:0005576~extracellular region	54	0.272	2.49E-20	S100A7/MMP2/CXCL12/CTGF/TGFBI/LOX/LOXL1/PMCH/CST3/HLA-C/SERPING1/MMP13/CD163/PTHLH/C1QA/C1QB/CHRDL1/GHRH/SERPINF2/COL1A2/TMSB4X/VCAN/FMOD/C3/LUM/COL3A1/CLU/DCN/ISLR/COL7A1/COL6A5/COL6A3/PYCARD/COL6A1/LAMB1/THBS2/SRGN/FN1/BMP4/COL4A2/COL4A1/RNASE6/ANXA1/COL15A1/SPARC/CCL16/COL5A2/LAMA4/FBLN1/COL14A1/APOL1/FBLN2/FBLN5/CD14
CC	GO:0070062~extracellular exosome	65	0.327	1.09E-16	S100A7/CD53/HLA-DMA/CXCL12/ACTR2/DAB2/CD44/TGFBI/CAP1/DPP7/GOLM1/FTL/BST2/SPARCL1/HLA-A/CST3/HLA-C/SERPING1/HLA-B/HLA-E/C1QA/STOM/C1QB/GNAQ/GNB1/SERPINF2/VAMP8/COL1A2/PDGFRB/SH3GL2/FRK/GNAI3/C3/IFITM3/LUM/CLU/ITGB2/ARPC5/SERPINH1/PCOLCE/ISLR/VCAM1/ARPC2/COL6A3/COL6A1/LAMB1/FN1/COL4A2/LGALS1/RNASE6/ITGA1/ANXA1/COL15A1/FZD4/PSMB9/ANXA2/LAMA4/FBLN1/COL14A1/FBLN2/FBLN5/FCGR2A/FABP5/CD14/CDH11
CC	GO:0005615~extracellular space	45	0.226	1.49E-16	FMOD/C3/LUM/LY86/CLU/COL3A1/DCN/MMP2/CXCL12/SERPINH1/PCOLCE/VCAM1/COL7A1/CTGF/TGFBI/COL6A3/LOX/LAMB1/LOXL1/GOLM1/SRGN/FN1/BMP4/SPARCL1/LGALS1/ANXA1/CST3/COL15A1/SERPING1/SPARC/CCL16/MMP13/ANXA2/PTHLH/STOM/AFP/FBLN1/COL14A1/APOL1/GHRH/SERPINF2/FBLN5/COL1A2/VCAN/CD14
CC	GO:0009986~cell surface	29	0.146	2.30E-15	CLU/ITGB2/LPAR1/CD53/HLA-DMA/GHRHR/VCAM1/CD44/CD93/FCER1G/HLA-DPB1/CSF1R/TYROBP/BST2/LGALS1/ANXA1/HLA-A/TSPAN14/ITGA1/HLA-C/SPARC/HLA-B/HLA-E/FZD4/ANXA2/HLA-F/SERPINF2/PDGFRB/HLA-DPA1
CC	GO:0005578~proteinaceous	22	0.111	3.07E-15	BMP4/FMOD/SPARCL1/LUM/LGALS1/COL15A1/SPARC/MMP2/MMP13/COL

	extracellular matrix				5A2/FBLN1/COL14A1/CTGF/COL6A5/FBLN2/FBLN5/COL6A3/TGFBI/COL1A2/VCAN/LOX/FN1
CC	GO:0005581~collagen trimer	15	0.075	1.59E-14	COL3A1/COL15A1/MMP13/COL5A2/SERPINH1/PCOLCE/C1QA/C1QB/COL14A1/COL7A1/COL6A5/COL6A3/COL1A2/COL6A1/LOX
CC	GO:0005886~plasma membrane	70	0.352	2.89E-11	QRFPR/ADCY8/LY86/TACR1/CD53/LPAR1/MMP2/GHRHR/DAB2/CD44/CD93/GPR45/CTGF/CXCR6/TGFBI/S1PR5/MC5R/CAP1/PTGER2/PTGER4/BST2/HLA-A/HLA-C/HLA-B/HLA-E/HLA-G/CD163/HLA-F/GRM3/GNAQ/GNB1/LPAR6/VAMP8/PDGFRB/HLA-DPA1/SH3GL2/HLA-DQB1/C3AR1/GNAI3/SNAP91/DRD3/C3/IFITM2/IFITM3/DRD5/FFAR1/OXTR/ITGB2/GNG11/GPR143/VCAM1/FCER1G/CD4/HLA-DPB1/TYROBP/CSF1R/SYT11/ITGA1/ANXA1/TSPAN14/SPARC/FZD4/ANXA2/GNGT1/CYBB/FCGR2B/FCGR2A/CD14/ADRA1D/CDH11
CC	GO:0071556~integral component of lumenal side of endoplasmic reticulum membrane	9	0.045	4.93E-11	HLA-DQB1/HLA-A/HLA-C/HLA-DPA1/HLA-B/HLA-DPB1/HLA-E/HLA-G/HLA-F
CC	GO:0042612~MHC class I protein complex	7	0.035	1.02E-10	HLA-DQB1/HLA-A/HLA-C/HLA-B/HLA-E/HLA-G/HLA-F
CC	GO:0005604~basement membrane	11	0.055	6.71E-10	LAMA4/FBLN1/COL4A1/COL7A1/TGFBI/CST3/SPARC/LAMB1/THBS2/LOXL1/ANXA2
CC	GO:0005788~endoplasmic reticulum lumen	14	0.070	4.50E-09	COL4A2/COL4A1/COL3A1/COL15A1/COL5A2/SERPINH1/COLGALT2/COL14A1/COL7A1/COL6A3/COL1A2/COL6A1/CD4/RCN1
CC	GO:0012507~ER to Golgi transport vesicle membrane	9	0.045	7.41E-09	HLA-DQB1/HLA-A/HLA-C/HLA-DPA1/HLA-B/HLA-DPB1/HLA-E/HLA-G/HLA-F
CC	GO:0005887~integral component of plasma membrane	34	0.171	1.50E-08	QRFPR/C3AR1/DRD3/TACR1/DRD5/FFAR1/OXTR/CD53/LPAR1/LAPTM5/CD44/CXCR6/FCER1G/MC5R/GOLM1/TYROBP/CSF1R/PTPRB/PTGER2/BST2/PTGER4/SYT11/HLA-A/TSPAN14/HLA-C/HLA-B/FZD4/CD163/STOM/GRM3/CYBB/LPAR6/HLA-DPA1/ADRA1D

CC	GO:0042613~MHC class II protein complex	7	0.035	1.54E-08	HLA-DQB1/HLA-A/HLA-C/HLA-DPA1/HLA-DPB1/HLA-DMB/HLA-DMA
CC	GO:0005765~lysosomal membrane	15	0.075	4.07E-08	HLA-DQB1/GNAI3/IFITM3/HLA-DMB/GPR143/HLA-DMA/ANXA2/DAB2/LAPTM5/GNAQ/GNB1/VAMP8/COL6A1/HLA-DPA1/HLA-DPB1
CC	GO:0031093~platelet alpha granule lumen	8	0.040	2.57E-07	ISLR/SERPINF2/CLU/SERPING1/TMSB4X/SPARC/SRGN/FN1
CC	GO:0030670~phagocytic vesicle membrane	7	0.035	7.28E-06	CYBB/HLA-A/HLA-C/HLA-B/HLA-E/HLA-G/HLA-F
MF	GO:0005201~extracellular matrix structural constituent	14	0.070	8.59E-15	COL4A2/COL4A1/LUM/COL3A1/COL15A1/COL5A2/FBLN1/LAMA4/COL14A1/FBLN2/COL1A2/VCAN/CD4/LAMB1
MF	GO:0005518~collagen binding	13	0.065	6.54E-14	COL14A1/CD44/SPARCL1/LUM/TGFBI/ITGA1/DCN/SPARC/MMP13/SERPINH1/PCOLCE/SRGN/FN1
MF	GO:0042605~peptide antigen binding	8	0.040	2.38E-09	HLA-DQB1/HLA-A/HLA-C/HLA-DPA1/HLA-B/HLA-DPB1/HLA-E/HLA-F
MF	GO:0048407~platelet-derived growth factor binding	5	0.025	1.43E-06	COL4A1/COL3A1/COL1A2/PDGFRB/COL6A1
MF	GO:0032395~MHC class II receptor activity	5	0.025	5.76E-06	HLA-DQB1/HLA-C/HLA-DPA1/HLA-DPB1/HLA-DMA
KEGG_PATHWAY	hsa05332:Graft-versus-host disease	11	0.055	8.93E-12	HLA-DQB1/HLA-A/HLA-C/HLA-DPA1/HLA-B/HLA-DPB1/HLA-DMB/HLA-E/HLA-DMA/HLA-G/HLA-F
KEGG_PATHWAY	hsa04145:Phagosome	18	0.091	9.56E-12	HLA-DQB1/NCF2/C3/HLA-A/ITGB2/HLA-C/HLA-B/HLA-DMB/HLA-E/HLA-DMA/HLA-G/HLA-F/FCGR2B/HLA-DPA1/FCGR2A/HLA-DPB1/THBS2/CD14
KEGG_PATHWAY	hsa05330:Allograft rejection	11	0.055	3.21E-11	HLA-DQB1/HLA-A/HLA-C/HLA-DPA1/HLA-B/HLA-DPB1/HLA-DMB/HLA-E/HLA-DMA/HLA-G/HLA-F
KEGG_PATHWAY	hsa05150:Staphylococcus aureus	12	0.060	8.96E-11	HLA-DQB1/C1QA/C3AR1/C1QB/FCGR2B/C3/HLA-DPA1/ITGB2/FCGR2A/HLA-

	infection				DPB1/HLA-DMB/HLA-DMA
KEGG_PATHWAY	hsa04512:ECM-receptor interaction	14	0.070	9.66E-11	COL4A2/COL4A1/COL3A1/ITGA1/COL5A2/LAMA4/CD44/COL6A5/COL6A3/COL1A2/COL6A1/LAMB1/THBS2/FN1
KEGG_PATHWAY	hsa04940:Type I diabetes mellitus	11	0.055	1.28E-10	HLA-DQB1/HLA-A/HLA-C/HLA-DPA1/HLA-B/HLA-DPB1/HLA-DMB/HLA-E/HLA-DMA/HLA-G/HLA-F
KEGG_PATHWAY	hsa05416:Viral myocarditis	12	0.060	1.67E-10	HLA-DQB1/HLA-A/HLA-C/HLA-DPA1/ITGB2/HLA-B/HLA-DPB1/HLA-DMB/HLA-E/HLA-DMA/HLA-G/HLA-F
KEGG_PATHWAY	hsa05320:Autoimmune thyroid disease	11	0.055	1.24E-09	HLA-DQB1/HLA-A/HLA-C/HLA-DPA1/HLA-B/HLA-DPB1/HLA-DMB/HLA-E/HLA-DMA/HLA-G/HLA-F
KEGG_PATHWAY	hsa04612:Antigen processing and presentation	12	0.060	4.20E-09	HLA-DQB1/HLA-A/HLA-C/CD4/HLA-DPA1/HLA-B/HLA-DPB1/HLA-DMB/HLA-E/HLA-DMA/HLA-G/HLA-F
KEGG_PATHWAY	hsa04514:Cell adhesion molecules (CAMs)	15	0.075	4.93E-09	HLA-DQB1/HLA-A/ITGB2/HLA-C/HLA-B/HLA-DMB/HLA-E/HLA-DMA/HLA-G/HLA-F/VCAM1/CD4/VCAN/HLA-DPA1/HLA-DPB1
KEGG_PATHWAY	hsa04151:PI3K-Akt signaling pathway	20	0.101	1.18E-07	COL4A2/COL4A1/COL3A1/ITGA1/GNG11/LPAR1/COL5A2/GNGT1/LAMA4/COL6A5/GNB1/LPAR6/COL6A3/COL1A2/PDGFRB/COL6A1/LAMB1/THBS2/FN1/CSF1R
KEGG_PATHWAY	hsa05200:Pathways in cancer	21	0.106	1.89E-07	BMP4/COL4A2/PTGER2/COL4A1/GNAI3/PTGER4/ADCY8/GNG11/LPAR1/MMP2/FZD4/CXCL12/GNGT1/LAMA4/GNAQ/GNB1/LPAR6/PDGFRB/LAMB1/FN1/CSF1R
KEGG_PATHWAY	hsa05166:HTLV-I infection	17	0.086	2.22E-07	EGR1/HLA-DQB1/ADCY8/HLA-A/HLA-C/ITGB2/HLA-B/HLA-DMB/HLA-E/HLA-DMA/FZD4/HLA-G/HLA-F/VCAM1/PDGFRB/HLA-DPA1/HLA-DPB1
KEGG_PATHWAY	hsa04974:Protein digestion and absorption	11	0.055	2.37E-07	COL4A2/COL14A1/COL4A1/COL7A1/COL6A5/COL3A1/COL6A3/COL1A2/COL15A1/COL6A1/COL5A2
KEGG_PATHWAY	hsa05146:Amoebiasis	11	0.055	1.37E-06	COL4A2/LAMA4/COL4A1/GNAQ/COL3A1/COL1A2/ITGB2/LAMB1/COL5A2/CD14/FN1
KEGG_PATHWAY	hsa04510:Focal adhesion	14	0.070	3.35E-06	COL4A2/COL4A1/COL3A1/ITGA1/COL5A2/LAMA4/COL6A5/COL6A3/COL1A2

					/COL6A1/PDGFRB/LAMB1/THBS2/FN1
KEGG_PATHWAY	hsa05140:Leishmaniasis	9	0.045	4.36E-06	HLA-DQB1/NCF2/C3/HLA-DPA1/ITGB2/FCGR2A/HLA-DPB1/HLA-DMB/HLA-DMA
KEGG_PATHWAY	hsa05133:Pertussis	9	0.045	6.62E-06	C1QA/C1QB/GNAI3/C3/PYCARD/IRF1/SERPING1/ITGB2/CD14
KEGG_PATHWAY	hsa05168:Herpes simplex infection	12	0.060	3.19E-05	HLA-DQB1/C3/HLA-A/HLA-C/HLA-DPA1/HLA-B/HLA-DPB1/HLA-DMB/HLA-E/HLA-DMA/HLA-G/HLA-F
KEGG_PATHWAY	hsa05169:Epstein-Barr virus infection	10	0.050	3.55E-05	HLA-DQB1/CD44/HLA-A/HLA-C/HLA-DPA1/HLA-B/HLA-DPB1/HLA-E/HLA-G/HLA-F
KEGG_PATHWAY	hsa05310:Asthma	6	0.030	4.42E-05	HLA-DQB1/FCER1G/HLA-DPA1/HLA-DPB1/HLA-DMB/HLA-DMA
KEGG_PATHWAY	hsa04611:Platelet activation	10	0.050	5.87E-05	GNAI3/GNAQ/VAMP8/ADCY8/COL3A1/COL1A2/FCER1G/FCGR2A/COL5A2/LCP2
KEGG_PATHWAY	hsa04080:Neuroactive ligand-receptor interaction	14	0.070	7.95E-05	C3AR1/PTGER2/DRD3/PTGER4/DRD5/TACR1/OXTR/LPAR1/GHRHR/GRM3/LPAR6/S1PR5/MC5R/ADRA1D
KEGG_PATHWAY	hsa05152:Tuberculosis	11	0.055	1.26E-04	HLA-DQB1/FCGR2B/C3/FCER1G/HLA-DPA1/ITGB2/FCGR2A/HLA-DPB1/HLA-DMB/HLA-DMA/CD14
KEGG_PATHWAY	hsa04672:Intestinal immune network for IgA production	6	0.030	3.97E-04	HLA-DQB1/HLA-DPA1/HLA-DPB1/HLA-DMB/HLA-DMA/CXCL12
KEGG_PATHWAY	hsa05322:Systemic lupus erythematosus	9	0.045	4.26E-04	HLA-DQB1/C1QA/C1QB/C3/HLA-DPA1/FCGR2A/HLA-DPB1/HLA-DMB/HLA-DMA
KEGG_PATHWAY	hsa05145:Toxoplasmosis	8	0.040	6.75E-04	HLA-DQB1/LAMA4/GNAI3/HLA-DPA1/HLA-DPB1/HLA-DMB/LAMB1/HLA-DMA
KEGG_PATHWAY	hsa05323:Rheumatoid arthritis	7	0.035	1.15E-03	HLA-DQB1/HLA-DPA1/ITGB2/HLA-DPB1/HLA-DMB/HLA-DMA/CXCL12
KEGG_PATHWAY	hsa04610:Complement and coagulation cascades	6	0.030	2.30E-03	C1QA/C3AR1/C1QB/C3/SERPINF2/SERPING1
KEGG_PATHWAY	hsa04724:Glutamatergic synapse	7	0.035	4.29E-03	GNGT1/GRM3/GNAI3/GNAQ/GNB1/ADCY8/GNG11
KEGG_PATHWAY	hsa04144:Endocytosis	10	0.050	5.07E-03	DAB2/ARPC2/HLA-A/HLA-C/HLA-B/ARPC5/HLA-E/SH3GL2/HLA-G/HLA-F

KEGG_PATHWAY	hsa04728:Dopaminergic synapse	7	0.035	7.52E-03	GNGT1/GNAI3/DRD3/GNAQ/GNB1/DRD5/GNG11
KEGG_PATHWAY	hsa04380:Osteoclast differentiation	7	0.035	8.39E-03	CYBB/FCGR2B/NCF2/FCGR2A/CSF1R/LCP2/TYROBP
KEGG_PATHWAY	hsa04713:Circadian entrainment	6	0.030	9.03E-03	GNGT1/GNAI3/GNAQ/GNB1/ADCY8/GNG11
KEGG_PATHWAY	hsa05321:Inflammatory bowel disease (IBD)	5	0.025	1.07E-02	HLA-DQB1/HLA-DPA1/HLA-DPB1/HLA-DMB/HLA-DMA
KEGG_PATHWAY	hsa04723:Retrograde endocannabinoid signaling	6	0.030	1.16E-02	GNGT1/GNAI3/GNAQ/GNB1/ADCY8/GNG11
KEGG_PATHWAY	hsa04062:Chemokine signaling pathway	8	0.040	1.26E-02	GNGT1/GNAI3/GNB1/ADCY8/CXCR6/GNG11/CCL16/CXCL12
KEGG_PATHWAY	hsa04725:Cholinergic synapse	6	0.030	1.69E-02	GNGT1/GNAI3/GNAQ/GNB1/ADCY8/GNG11
KEGG_PATHWAY	hsa04670:Leukocyte transendothelial migration	6	0.030	1.94E-02	VCAM1/GNAI3/NCF2/ITGB2/CXCL12/MMP2
KEGG_PATHWAY	hsa04015:Rap1 signaling pathway	8	0.040	2.31E-02	GNAI3/GNAQ/ADCY8/PDGFRB/ITGB2/LPAR1/CSF1R/LCP2
KEGG_PATHWAY	hsa05222:Small cell lung cancer	5	0.025	2.76E-02	COL4A2/LAMA4/COL4A1/LAMB1/FN1
KEGG_PATHWAY	hsa04727:GABAergic synapse	5	0.025	2.76E-02	GNGT1/GNAI3/GNB1/ADCY8/GNG11
KEGG_PATHWAY	hsa04640:Hematopoietic cell lineage	5	0.025	2.97E-02	CD44/ITGA1/CD4/CD14/CSF1R
KEGG_PATHWAY	hsa04540:Gap junction	5	0.025	3.09E-02	GNAI3/GNAQ/ADCY8/PDGFRB/LPAR1
KEGG_PATHWAY	hsa04020:Calcium signaling pathway	7	0.035	3.38E-02	GNAQ/ADCY8/TACR1/DRD5/PDGFRB/OXTR/ADRA1D
KEGG_PATHWAY	hsa05032:Morphine addiction	5	0.025	3.43E-02	GNGT1/GNAI3/GNB1/ADCY8/GNG11
KEGG_PATHWAY	hsa05134:Legionellosis	4	0.020	3.61E-02	C3/PYCARD/ITGB2/CD14
