# Supplementary figures and images for: Identification of the Potential Molecular Mechanism of TGFBI Gene in Persistent Atrial Fibrillation
Source: Comput Math Methods Med. 2022 Nov 8;2022:1643674. doi: 10.1155/2022/1643674 (PMC9666036; doi:10.1155/2022/1643674)

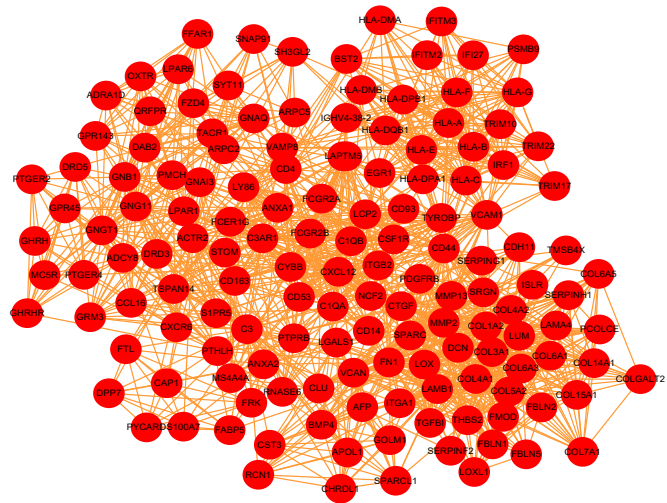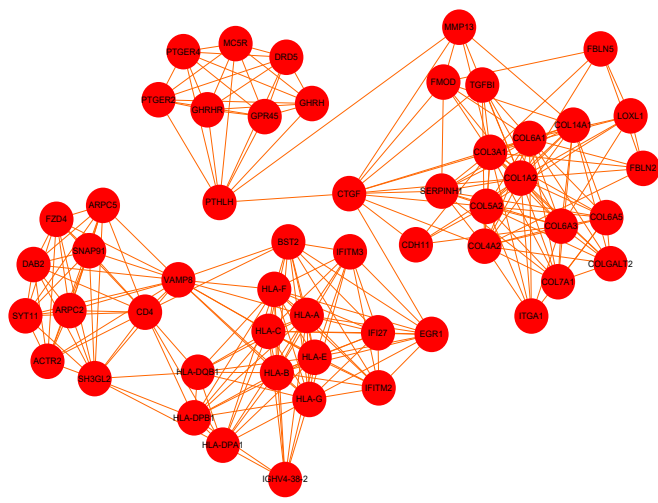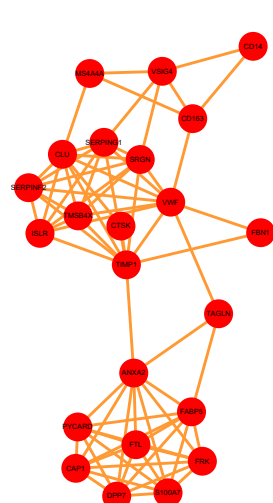

Supplement: Supplementary Materials — Table S1: GO annotation of TGFBI and coexpression genes enriched in AF. Table S2: GO annotation and KEGG pathway TGFBI and coexpression genes enriched in cluster 1. Figure S1: the clusters constructed from the PPI-network. [file 1643674.f1.zip › Figure S1.pdf]
